# MicroRNAs as potent regulators in nitrogen and phosphorus signaling transduction and their applications

**DOI:** 10.1007/s44154-024-00181-x

**Published:** 2024-09-12

**Authors:** Yuzhang Yang, Yanting Liang, Chun Wang, Yanwei Wang

**Affiliations:** State Key Laboratory of Tree Genetics and Breeding, National Engineering Research Center of Tree Breeding and Ecological Restoration, Key Laboratory of Genetics and Breeding in Forest Trees and Ornamental Plants, Ministry of Education, The Tree and Ornamental Plant Breeding and Biotechnology Laboratory of National Forestry and Grassland Administration, College of Biological Sciences and Biotechnology, Beijing Forestry University, Beijing, 100083 China

**Keywords:** miRNA, ncRNA, Plant, Nitrogen, Phosphorus, Signaling

## Abstract

Nitrogen (N) and phosphorus (Pi) are essential macronutrients that affect plant growth and development by influencing the molecular, metabolic, biochemical, and physiological responses at the local and whole levels in plants. N and Pi stresses suppress the physiological activities of plants, resulting in agricultural productivity losses and severely threatening food security. Accordingly, plants have elaborated diverse strategies to cope with N and Pi stresses through maintaining N and Pi homeostasis. MicroRNAs (miRNAs) as potent regulators fine-tune N and Pi signaling transduction that are distinct and indivisible from each other. Specific signals, such as noncoding RNAs (ncRNAs), interact with miRNAs and add to the complexity of regulation. Elucidation of the mechanisms by which miRNAs regulate N and Pi signaling transduction aids in the breeding of plants with strong tolerance to N and Pi stresses and high N and Pi use efficiency by fine-tuning *MIR* genes or miRNAs. However, to date, there has been no detailed and systematic introduction and comparison of the functions of miRNAs in N and Pi signaling transduction from the perspective of miRNAs and their applications. Here, we summarized and discussed current advances in the involvement of miRNAs in N and Pi signaling transduction and highlighted that fine-tuning the *MIR* genes or miRNAs involved in maintaining N and Pi homeostasis might provide valuable sights for sustainable agriculture.

**Supplementary Information:**

The online version contains supplementary material available at 10.1007/s44154-024-00181-x.

## Introduction

Plants require 14 essential mineral elements (Marschner 1995), among which, nitrogen (N) and phosphorus (Pi), the two most critical major elements, play an irreplaceable role in the growth and development of plants. Plants inevitably suffer from N and Pi deficiency, while the production of N fertilizer demands high energy consumption and phosphate rock faces the risk of exhaustion as the source of Pi fertilizer. Therefore, it is urgently needed to cultivate plants with strong tolerance to N and Pi stresses or high N use efficiency (NUE) and Pi use efficiency (PUE) by molecular design to achieve sustainable agriculture. In the past few years, great progress has been made in identifying participators of N and Pi signaling transduction. Signals such as sensors, transporters, transcription factors (TFs), hormones, and non-coding RNAs (ncRNAs) form sophisticated N and Pi signaling transduction pathways in plants. Their constitutive expression and mutual effects contribute to the maintenance of N and Pi homeostasis in plants systematically or locally (Gao et al. 2022; Krapp et al. 2014). Several recent reviews also summarize the signals in the N-starvation response (NSR), primary nitrate response (PNR), and Pi starvation (PS) rescue system (PSR) of plants (Paz-Ares et al. 2022; Shanks et al. 2024; Zhang et al. 2023).

NcRNAs are generally categorized as small RNAs (sRNAs) (18–200 nt) and long non-coding RNAs (lncRNAs) (> 200 nt). Among them, two main classes of sRNAs are microRNAs (miRNAs) and small interfering RNAs (siRNAs) that regulate numerous biological processes through transcriptional and post-transcriptional gene silencing. Plant *MIR* genes encode the primary transcripts (pri-miRNAs) with the participation of DNA-dependent RNA polymerase II (Pol II) and further form secondary transcripts with stem-loop structure (pre-miRNAs) through self-complementary base pairing (Xie et al. 2005). The dsRNA-binding protein HYPONASTIC LEAVES1 (HYL1) and C2H2 zinc finger protein SERRATE (SE) bind to DICER-LIKE1 (DCL1) to form nuclear dicing bodies (D-bodies) (Dong et al. 2008; Kurihara et al. 2006). After the two-step cleavage by D-bodies and HEN1-mediated methylation, the double-stranded miRNAs (miRNA/miRNA* duplex) are formed (Park et al. 2002; Xie et al. 2003). The duplex is transported into the cytoplasm through the HASTY (HST) protein. Mature miRNAs are finally produced and bind to Argonaute (AGO) proteins to form the RNA-induced silencing complexes while miRNA*s degrade (Baumberger and Baulcombe 2005), which guide the cleavage of targets in the cytoplasm (Borges and Martienssen 2015; Chen 2005). An array of proteins have been discovered to regulate miRNA transcription and pri-miRNA processing and modification, such as SMALL1 and TREX-2 (Li et al. 2018; Zhang et al. 2020). Based on different biogenesis modes and action mechanisms, siRNAs are double-stranded RNAs produced from perfectly complementary long double-stranded RNAs (dsRNAs) (Zhan and Meyers 2023). Different from sRNAs, plant lncRNAs typically possess an mRNA-like single-stranded structure with a 5' m^7^G cap and a 3' poly (A) tail, and are classified as long intergenic non-coding RNAs, long non-coding natural antisense transcripts, long intronic non-coding RNAs, and overlapping lncRNAs (Yang et al. 2023). Circular RNAs (circRNAs) as a novel type of lncRNAs have a covalently closed loop structure. They mainly arise from exons (exonic circRNAs) or introns (intronic circRNAs) through back splicing (Patop et al. 2019).

Recently, emerging investigations have shown that the differential changes in the abundance of ncRNAs modulate the expression of corresponding genes in N and Pi signaling transduction (Jamla et al. 2023; Song et al. 2019; Wang et al. 2017a, b). Integrating the role of miRNAs in N and Pi signaling transduction could provide fundamental insights into maintaining N and Pi homeostasis by molecular breeding via miRNAs. However, so far, there have been few detailed and systematic summarizations and comparisons of the functions of miRNAs in regulating N and Pi signaling transduction from the perspective of ncRNAs and their applications.

In this review, we comprehensively summarized and compared the basic research and development of N and Pi signaling transduction and miRNAs-mediated regulation of N and Pi signaling transduction and their properties, and expanded the RNA-RNA interactions affecting miRNA-mediated regulation of N and Pi signaling transduction. Furthermore, we provided a current understanding concerning the molecular and physiological roles of well-studied miRNAs in maintaining plant N and Pi homeostasis and discussed the widely-used miRNA methods and techniques, which would provide candidate genes and technical support for the breeding of plants with high NUE and PUE or strong tolerance to N and Pi stresses. This review might provide a strategic sight into sustainable agriculture via fine-tuning *MIR* genes or miRNAs.

## Nitrogen and phosphorus signaling transduction

**Fig. 1:**
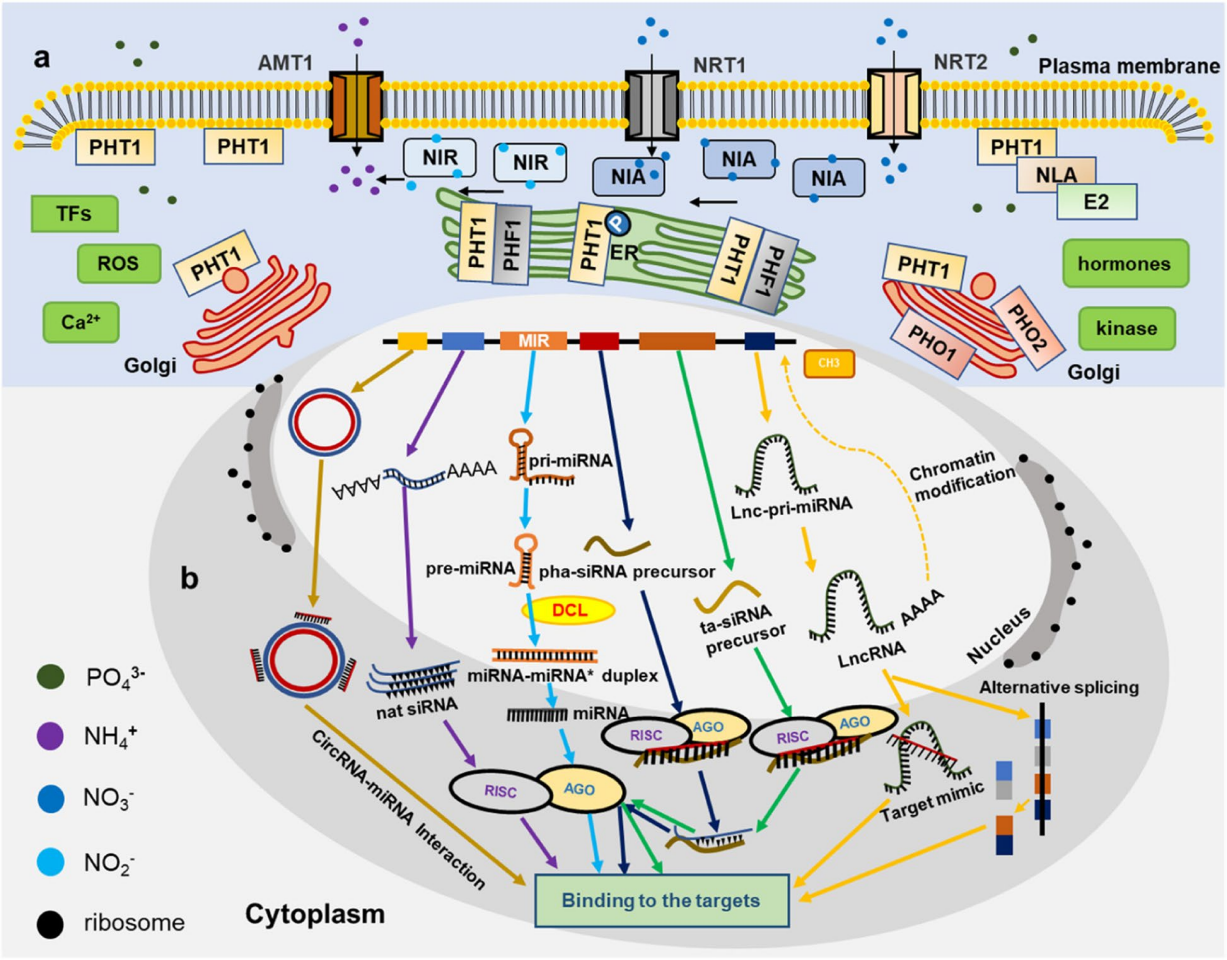
Well-characterized N and Pi molecular components. **a** Transporters are the enforcers of N and Pi signaling transduction in plants. The signals regulate the activity and content of N and Pi transporters to interweave into the complex signaling transduction pathways. The dark green circle represents PO_4_^3−^, the purple circle represents NH_4_^+^, the dark blue circle represents NO_3_^−^, the blue circle represents nitrite (NO_2_^−^), and the black circle represents ribosomes. **b** The miRNAs and other ncRNAs regulate the expression of targets to activate the regulation of N and Pi signaling transduction. Different colored lines with arrows represent the regulations of ncRNAs involved in the N and Pi signaling transduction

So far, well-characterized molecular components defined as signals of the primary nitrate response (PNR), N-starvation response (NSR), and Pi starvation (PS) rescue system (PSR) have been proved to regulate the sensing, acquisition, and transportation of N and Pi nutrition (Fig. 1). The components and their roles are briefly summarized and discussed below.

### N signaling transduction

#### The transporters involved in N sensing and acquisition

Nitrate (NO_3_^−^) and ammonium (NH_4_^+^) are preferred forms of N nutrition for plant growth and development, which are deficient in agricultural fields, especially forest soils. NO_3_^−^ transporters of plants are divided into nitrate transporter 1/peptide transporter family (NRT1/PTR or NPF), nitrate transporter 2 family (NRT2), chloride channel family (CLC), and slow anion channel-associated homologues (SLAC/SLAH), and the root-specific transporters are well-studied (Fig. 1a). In Arabidopsis, NO_3_^−^ uptake is mediated by a high-affinity transport system primarily consisting of NRT2 transporters due to NO_3_^−^ concentrations generally less than 1 mmol L^−1^ in soil (Garnett et al. 2003). The functional unit of high-affinity NO_3_^−^ inflow may be a tetramer composed of NRT2.1 and NAR2.1 (Yong et al. 2010). However, the function of the formation has not been elucidated to date. In addition, a dual-affinity transporter AtNPF6.3/AtCHL1/AtNRT1.1 acts as a transceptor (transporter and receptor) with amphiphilic structures switching between phosphorylation and dephosphorylation at Thr101, a threonine residue, which contributes to both high-affinity and low-affinity NO_3_^−^ uptake (Bouguyon et al. 2015; Ho et al. 2009). Unlike NPF6.3, ATNPF4.6 is a pure low-affinity NO_3_^−^ transporter (Huang et al. 1999). In rice, OsNPF2.4 is also involved in the low-affinity NO_3_^−^ acquisition, transportation of NO_3_^−^ from roots to stems, and regulation of N mobilization from source to sink organs (Xia et al. 2014).

Moreover, NO_3_^−^ is converted to NO_2_^−^ by nitrate reductases encoded by NIA1 and NIA2, and NO_2_^−^ is converted to NH_4_^+^ with the aid of nitrite reductase encoded by NIR. In Arabidopsis, six AMT-type transporters manipulate the functions of sensing and transporting NH_4_^+^ (Yuan et al. 2007). Subsequent determination and comparison of NH_4_^+^ uptake rates in transgenic Arabidopsis with single knockout, double knockout, and multiple knockout ATMs prove that ATM1 plays a dominant role in NH_4_^+^ uptake and ATMs have cooperative functions (Loqué et al. 2006; Yuan et al. 2007). Overall, these N transporters act as the most fundamental N signals in sensing and transporting N nutrition.

#### The N transporter regulation

Correspondingly, the expression of the NO_3_^−^ transporters is regulated by transcription factors (TFs), such as NLP6, NLP7, SPL9, STOP1, TCP20, and BES1 (Cheng et al. 2023; Wang et al. 2023b; Tokizawa et al. 2023; Ye et al. 2021; Zhang and Forde 1998). In rice, the NRT1.1 receptor activates NLP TFs that play a central role in the regulation of PNR and fine-tune the expression of NRT2.1 (Hu et al. 2019; Ueda and Yanagisawa 2023). Thus, NLP and NRT form a regulatory cascade of N sensing and transport. Recent studies show that new TFs regulate the expression of NO_3_^−^ transporters. For instance, *NAC1* can regulate the expression of NRT2.4 in apples (Wang et al. 2024). TFs also can regulate the expression of AMTs. For instance, indeterminate domain 10 (OsIDD10) regulates the expression of *OsAMT1;2*. Despite the decrease in the transcript abundance of *OsAMT1;2*, the effect of NH_4_^+^ on OsIDD10 knockout mutants is still present, which indicates other TFs should regulate the transcript abundance of AMT1;2 (Xuan et al. 2013). Interestingly, Long Hypocotyles 5 (*HY5*) negatively regulates *OsAMT1;2* transcript abundance (Huang et al. 2015), and *OsDOF18* positively regulates the transcript abundance of *OsAMT1;1*, *OsAMT1;3*, *OsAMT2;1*, and *OsAMT4;1* (Wu et al. 2017). These results suggest that the expression of N transporters is regulated at the transcriptional level.

Additionally, protein phosphorylation, a universal post-transcriptional modification, regulates the activity of transporters. In Arabidopsis, the sensing and transport activity of the core NO_3_^−^ transporter AtNPF6.3 is determined by Thr101 phosphorylation and dephosphorylation, which are regulated by a calcium-dependent protein kinase (CIPK) (Bouguyon et al. 2015). In addition, high NH_4_^+^ supply inhibits the activity of AMT1;1 by phosphorylating Thr460 on the C-terminal trans-activating domain of AMT1;1 (Lanquar et al. 2009). Later, CIPK23 controls the phosphorylation of Thr460, specifically interacts with AMT1;1 and AMT1;2 in vivo, and negatively regulates AMT1-dependent N uptake (Straub et al. 2017). These results suggest post-translational regulation of AMTs might involve phosphorylation/dephosphorylation events triggered by complex N signal inputs, further indicating the complexity of N uptake. Thus, the regulation of the N transporter depends on phosphorylation. Besides, AtNRT1.7 is responsible for source-sink remobilization of NO_3_^−^ in leaves during N starvation (Fan et al. 2009). AtNRT1.7 interacts with the cyclic E3 ligase NLA on the plasma membrane and is ubiquitinated by NLA via the 26S proteasome pathway (Liu et al. 2017b). Under N restriction, the reduction of *NLA* expression increases the abundance of AtNRT1.7, enhancing the re-flow of NO_3_^−^ from source to destination. Thus, ubiquitination-mediated post-translational regulation also regulates N remobilization. Overall, the transcriptional and post-transcriptional regulations fine-tune the activity and content of N transporters.

#### Other signals that regulate N signaling transduction

Moreover, other signals also regulate N signaling transduction in plants, such as hormones (auxin, cytokinin, abscisic acid, etc.), ncRNAs, reactive oxygen species (ROS), chemicals, and secondary messenger molecules. Additionally, Ca^2+^ acts as a core secondary messenger in response to NO_3_^−^_._ NRT1.1/AtNPF6.3 and phospholipase C mediate the increase of calcium (Ca^2+^) in response to NO_3_^−^ required for changes in the expression of prototypical nitrate-responsive genes (Riveras et al. 2015). Subsequent studies further reveal that the calcium-sensor protein kinases (CPK) exist in plants and NO_3_^−^ could induce the accumulation of Ca^2+^ and the rapid nuclear translocation of CPK. These results suggest that Ca^2+^ can act as a secondary messenger in response to NO_3_^−^ (Liu et al. 2017a). Besides, NO also acts as a secondary messenger responding to the assimilation and absorption of NO_3_^−^. Thus, the secondary messenger molecules are indispensable N signals.

Recent research also finds a low Fe status substantially enhances protein N-glycosylation via a Vitamin C1 (VTC1)-independent pathway that reduces NH_4_^+^ efflux to increase NUE in Arabidopsis under elevated NH_4_^+^ supply (Li et al. 2024). This suggests that NH_4_^+^ uptake in roots is regulated by the feedback of plant endogenous N status. In summary, N signaling transduction is diverse. However, it is still unclear how N signal reprogramming process occurs after N transporters sense the N status.

### Pi signaling transduction

#### The transporters involved in Pi sensing and acquisition

Different from the characteristics of N nutrition that exists in almost unlimited resources in the atmosphere, Pi nutrition is a finite resource. Plants preferentially take up phosphate (PO_4_^3−^) as orthophosphate, an ion with low solubility and easy fixation in soil, making the restriction of PO_4_^3−^ a common condition. However, the signaling transduction of Pi is similar to that of N. Plants dissolve and release PO_4_^3−^ through the PSR, including phosphatases or nucleases, and specific phosphate transporters, such as high-affinity transporters (PHTs) and Pi/H^+^ transporters (Młodzińska and Zboińska 2016). PHT gene family members (PHT1, PHT2), as the most well-studied Pi transporters, have been demonstrated to regulate the sensing and absorption of PO_4_^3−^ in Arabidopsis, rice, wheat, and poplar (Ayadi et al. 2015; Sun et al. 2012; Zhang et al. 2016).

In Arabidopsis, high-affinity and low-affinity PO_4_^3−^ absorption systems are found. High-affinity systems have an affinity for PO_4_^3−^ at low concentrations, with *Km* values ranging from 3 to 10 µmol L^−1^, while low-affinity systems operate at high PO_4_^3−^ concentrations, with *Km* values ranging from 50 to 300 µmol L^−1^. The two systems are mediated by the PHT1 family (Ayadi et al. 2015). So far, AtPHT1;1–1;9 have been identified. Except for AtPHT1;6, the transcripts of the other eight AtPHTs are detected in roots, suggesting that the eight AtPHT1s may play a role in PO_4_^3−^ acquisition (Ayadi et al. 2015). Intriguingly, AtPHT1;5 is essential for mobilizing the PO_4_^3−^ from root to stem rather than directly from the soil (Nagarajan et al. 2011). Combinedly, root PO_4_^3−^ absorption is mediated by AtPHT1;1–1;4 and AtPHT1;7–1;9 protein.

#### The Pi transporter regulation

Similar to the N transporters, the Pi transporters are regulated at the transcriptional and post-transcriptional levels. There are core TFs that regulate Pi signaling transduction. AtPHR1 is the first identified PS regulator in higher plants and is homologous to Chlamydomonas reinhardtii PSR1 (Wykoff et al. 1999). PHR1, a member of the GARP family, contains a DNA-binding domain similar to MYB (Rubio et al. 2001). PHR1-like (PHL) TFs are subsequently identified in Arabidopsis, and the homologs have been found in rice (Zhou et al. 2008). Subsequent research suggests PHR1/PHL controls the expression of genes in PSR by binding to PHR1 binding sites (P1BS) (Ruan et al. 2015). With the dual stimulation of Pi starvation and jasmonic acid (JA) signaling, PHR1 positively regulates the expression of miR399 and PHF1. Moreover, PHOSPHATE2 (PHO2) can degrade Pi transporters and transporter facilitator1 (PHF1), and PHO1 also positively regulates Pi transporters (Kumar et al. 2017). Recently, TFs such as WRKY6, WRKY10, WRKY65, and NIGT1 are also confirmed to be involved in regulating the Pi transporter expression (Wang et al. 2023a; Yang et al. 2022; Zhang et al. 2023a). Overall, the transcriptional regulation of Pi transporters is a critical fulcrum in Pi signaling transduction.

Additionally, phosphorylation of the PHT1s on the endoplasmic reticulum (ER) weakens its binding to PHF1, enabling transportation to plasma membrane (PM) or degradation through the vesicles of Golgi (Fig. 1a). PHT1.1 contains at least two phosphorylation sites, namely S514 and S520 in Arabidopsis (Hem et al. 2007; Nühse et al. 2004). Under PO_4_^3−^ starvation, S514 is dephosphorylated, promoting the transport of AtPHT1.1 from the ER to the PM. S520 is phosphorylated by AtCK2, which increases its activity to absorb PO_4_^3−^. Together, the two pathways enhance the ability of Arabidopsis to absorb PO_4_^3−^. Conversely, at high PO_4_^3−^ concentration, S514 is phosphorylated, leaving more AtPHT1.1 in the ER. At the same time, S520 is dephosphorylated (Wang et al. 2020a). These pathways synergistically reduce its ability to absorb PO_4_^3−^. A similar pattern is also found in rice (Yang et al. 2020). Moreover, AtSnRK1-mediated phosphorylation of S11 in AtPHR1 negatively controls its transcriptional activity (Trejo-Fregoso et al. 2022). Interestingly, TFs critical to the regulation of Pi transporter expression are also regulated by protein phosphorylation. For instance, AtMPK3/6-mediated phosphorylation of AtWRKYs positively controls the expression of Pi transporters (Mao et al. 2011). These results show that protein phosphorylation is widespread in the regulation of Pi transporters.

#### Other signals that regulate Pi signaling transduction

Moreover, some systemic or local signals, such as hormones (auxin, cytokinin, strigolactone (SL), JA, etc.), ncRNAs, sugars, ROS, and secondary messenger molecules, are also activated to regulate Pi signaling transduction (Chevalier et al. 2019; Czarnecki et al. 2013; de Souza Campos et al. 2019; Muneer and Jeong 2015). Notably, since the initial identification of the SPX domain proteins and inositol polyphosphate molecules (InsPs) in the PSR, numerous studies have further elucidated their roles in regulating Pi signaling transduction. SPX with a 140—400 amino-acid-long domain is named after the suppressor of yeast gpa1 (Syg1), yeast cyclin-dependent kinase inhibitor phosphatase 81 (Pho81), and human Xenotropic and Polytropic Retrovirus receptor1 (Xpr1), and SPX proteins can affect intracellular ATP levels and act as the core sensor to maintain the stability of intracellular PO_4_^3−^ (Duan et al. 2008; Hürlimann et al. 2009; Guo et al 2023; Wang et al. 2009). Four SPX proteins containing the SPX domains have been characterized in plants. PHR1-interacting SPX proteins only contain the SPX domains, while PHO1, SPX-MFS vacuolar Pi influx transporters, and NLA additionally contain EXS, MFS, and RING domains with varying functions, respectively (Park et al. 2014; Wang et al. 2015a; Wild et al. 2016). In Arabidopsis, SPX1, SPX2, and SPX4 control PHR1, and their homologs have also been functionally characterized with varying roles in rice (Wang et al. 2014; Lv et al. 2014; Puga et al. 2014; Osorio et al. 2019). Subsequent research show that the SPX domain senses InsPs as a Pi-signaling repressor and the SPX protein regulates PHRs under Pi starvation in rice and willow (Ding et al. 2021; Liu et al. 2014; Guan et al. 2022; Ruan et al. 2019). And two E3 ligases (SDEL1 and SDEL2) are identified to control SPX4 degradation. These ligases directly interact with SPX4 and regulate the stability of SPX4 together with PHR2 to accommodate the availability of Pi nutrition (Ruan et al. 2019). Intriguingly, recent studies suggest that hormones, such as SLs (Gu et al. 2023), are involved in the regulation of SPX, which has increased the complexity of the Pi network in response to Pi fluctuations.

InsPs also have been reported to act as Pi signals in plants (Wild et al. 2016; Zhu et al. 2019). InsP6 is considered to be a storage molecule that accumulates in the form of phytic acid in plants (Secco et al. 2017). Phosphorylation of InsP6 is catalyzed by inositol phosphokinase ITPK1 to form InsP7 (Riemer et al. 2021). VIH1 and VIH2 (homologs of PPIP5K, diphosphoinositol polyphosphate) convert InsP7 to InsP8 or InsP6. The preferred ligand of SPX protein is InsP8, which could restore the interaction between SPX1 and PHR1 in vivo (Dong et al. 2019; Ried et al. 2021). Under Pi-repletion conditions, the activity of VIH1 and VIH2 is allosterically regulated by the high Pi, leading to the increased levels of InsP8 and the formation of the InsP8-SPX-PHR complex (Gu et al. 2019; Zhu et al. 2019). InsP8 binds to SPX proteins to stabilize the helix alpha1 structure, which allows InsPs to uncouple PHR protein dimers allosterically. The SPX protein can then interact with the MYB domain and prevent the PHR protein from binding to the promoter of the PSR genes (Ried et al. 2021). Interestingly, kinase activity and ATP levels are reduced in the absence of Pi, resulting in decreased levels of InsP8. The released PHR can deactivate the PSR genes to promote Pi uptake (Dong et al. 2019; Zhu et al. 2019). The role of InsPs and SPX in regulating Pi signals has been summarized recently (Satheesh et al. 2022). However, the structure of the SPX-InsP-PHR complex in Pi signaling transduction still needs to be further elucidated considering that Pi signaling transduction is complex.

### The interaction of N-Pi signaling transduction

Past knowledge is mostly limited to investigating the role of N and Pi signals under N or Pi stress respectively. However, N and Pi signaling transduction are not completely independent but interact with each other, showing a relationship of mutual promotion or antagonism (Paz-Ares et al. 2022; Zhong et al. 2023). So far, four different levels of N-Pi signaling crosstalk have been found, including the interaction between NRT1.1 and SPX4 (Hu et al. 2019), the interaction between NIGT1 and RLI1 TFs (Kiba et al. 2018), NRS signaling downregulating PSI genes through PHR1 and PHO2 (Medici et al. 2019), and the interaction between PS and AMT1 (Tian et al. 2021). These interactions have begun to address the equilibrium acquisition of N and Pi, which are regulated by miRNAs. These results suggest that miRNAs act as potent regulators of N and Pi signaling transduction. Thus, in this review, we updated the involvement of miRNAs in N and Pi signaling transduction from the perspective of miRNAs that alter a wide range of molecular, biochemical, structural, and physiological responses.

## The involvement of plant miRNAs in nitrogen and phosphorus signaling transduction

The involvement of miRNAs in N and Pi signaling transduction mainly includes miRNA-mediated regulation, their properties, such as the pleiotropy and mobility, and the RNA-RNA interactions.

### MiRNAs-mediated regulation of N and Pi signaling transduction

**Fig. 2:**
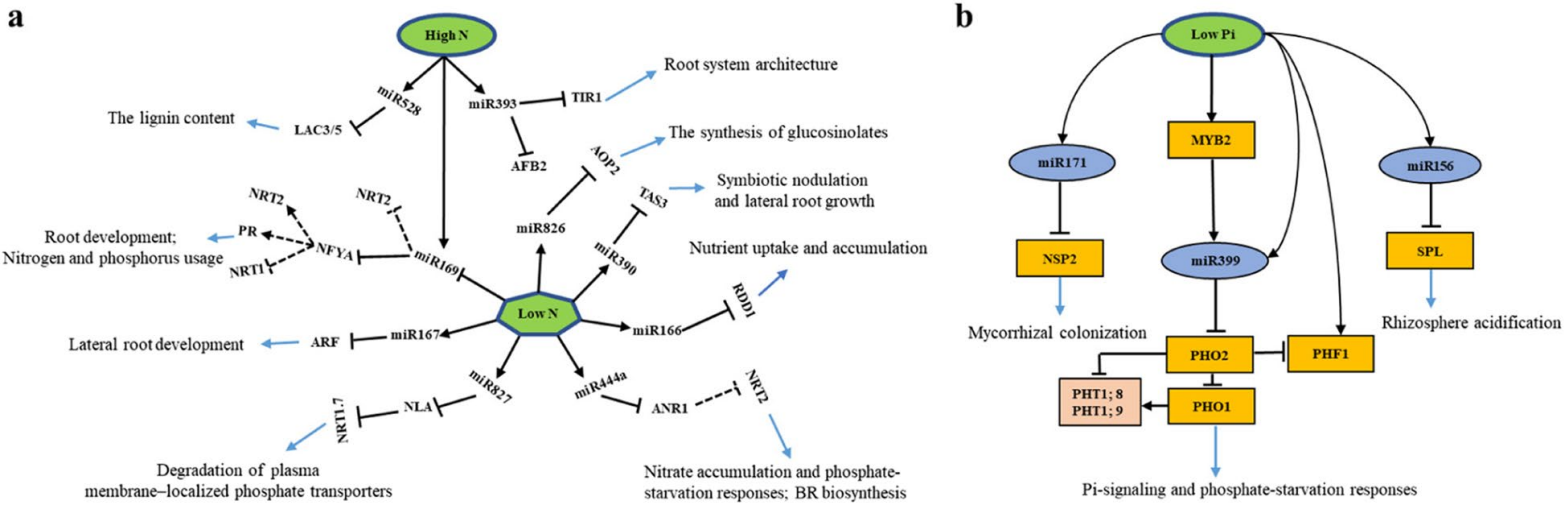
Plant miRNAs involved in N and Pi signaling transduction and their functions. **a** High N and low N affect the expression of plant miRNAs involved in N signaling transduction and their regulatory functions are described. **b** Low Pi affects the expression of plant miRNAs involved in Pi signaling transduction and their regulatory functions are described. The black line with an arrow represents the positive regulation, the black line with a smooth top represents the negative regulation, and the dotted line needs further verification. The blue arrows represent the biological functions affected by miRNAs

N and Pi stresses activated the synthesis and regulation of N and Pi transporters, the regulation of plant hormone biosynthesis, and reprogramming of gene expression. miRNAs play a critical role in N and Pi signaling transduction by regulating the biological processes (Table 1, Fig. 2).

**Table 1:** miRNAs-mediated regulation of N and Pi signaling transduction

Biological Function	ncRNAs	Target	Plant species	N	Pi	Source
Transporter protein expression	miR164	*NAC1*	apple	+	NE	Wang et al. 2024
	miR166	*RDD1*	rice	-	-	Iwamoto and Tagiri 2016
	miR167c	*ARF6*, *ARF8*	soybean	+	NE	Wang et al. 2015b
	miR169c	*NFYA-B1*	wheat	-	-	Qu et al. 2015
	miR169o	*NFYA*	rice	+	NE	Yu et al. 2018
	miR399	*PHO2*	Arabidopsis, rice	NE	+	Bari et al. 2006, de Souza Campos et al. 2019
	*PILNCR1*	miR399	maize	NE	-	Du et al. 2018
	miR826	*AOP2*	Arabidopsis	+	NE	He et al. 2014
	miR5090					
	miR827	*NLA*	Arabidopsis	+	+	Lin et al. 2013
Hormone biosynthesis	miR160	*ARF10*, *ARF16*, *ARF17*	*M. truncatula*	+	NE	Nizampatnam et al. 2015
	miR393	*AFB3*	Arabidopsis	+	NE	Vidal et al. 2010
	miR444	*MADS*	rice	+	+	Jiao et al. 2020, Yan et al. 2014
	miR390	*TAS3*	*M*. *truncatula*	+	NE	Hobecker et al. 2017
ROS scavenging capacity	miR528	*LAC3 LAC5*	mazie	-	NE	Sun et al. 2018
Plant-Rhizobium symbiosis	miR166	*HD-ZIP III*	*M. truncatula*	-	NE	Boualem et al. 2008
	miR167c	*ARF8*	soybean	+	NE	Wang et al. 2015b
	miR171c	*NSP2*	*L. Japonicus*	-	NE	De Luis et al. 2012
	miR171o	*SCL6*	soybean	-	NE	Hossain et al. 2019
	miR171q	*NSP2*				
	miR172	*AP2*	*L. japonicus,* soybean, and common bean	+	NE	Yan et al. 2013, Wang et al. 2014
	miR482	*NB-LRR*	soybean	NE	NE	Li et al. 2010
	miR1512	*CDB*				
	miR1515	*DCL*				
	miR1507	*NB-LRR*	*M. truncatula*	NE	NE	Sós Hegedűs et al. 2020
	miR2109					
	miR2118					
	miR2111	*TML*	*M*. *truncatula,* *L. Japonicus*	+	NE	Moreau et al. 2021, Okuma et al. 2020
Mycorrhizal colonization	miR171a-g	*NSP2*	*M. truncatula*	-	-	Lauressergues et al. 2012, Couzigou et al. 2017
Plant-environment interaction	miR156	*SPL*	Arabidopsis	+	+	Lei et al. 2016

*Abbreviations* *NAC*, NAM-ATAF1/2-CUC2, *RDD1* rice Dof daily fluctuations 1, *ARF* auxin response factor, *NFYA* Nuclear Factor Y subunit A, *PHO2* PHOSPHATE2, *AOP2* ALKENYL HYDROXALKYL PRODUCING2, *NLA* nitrogen limitation adaptation, *TAS* *trans-*acting siRNA, *AFB3* Auxin Signaling F-Box 3 receptor, *MADS* MIKCC-type MADS box, *LAC* *LACCASE3,* *HD-ZIP III* Class III homeodomain leucine zipper, *NSP2* Nodulation signaling pathway2, *SCL* SCARECROW-LIKE, *AP2* APETALA2, *NB-LRR* nucleotide-binding leucine-rich repeat, *CDB* Calmodulin-binding region, *DCL* Dicer-like protein, *TML* Too Much Love, *SPL* SQUAMOSA PROMOTER BINDING PROTEIN-LIKE *M truncatula* *Medicago truncatul,* *L japonicus, Lotus japonicus*

*‘ + ’ represents positive regulation of miRNAs in responding N or Pi stress, ‘-’ represents negative regulation of miRNAs in responding N or Pi stress, ‘NE’ represents no evidence*

#### MiRNAs involved in the regulation of N and Pi transporter

Most of miRNAs, such as miR167, miR169, and miR399, regulate the expression of N and Pi transporters by targeting TFs. For instance, in Arabidopsis, the promoter regions of *AtNRT1.1* and *AtNRT2.1* contain four and one “-CCAAT-” elements, respectively, which are Nuclear Factor-Y Subunit A (NFYA) binding regions. In transgenic Arabidopsis over-expressing miR169a, *AtNRT1.1* and *AtNRT2.1* expression are significantly reduced (Zhao et al. 2011), thereby reducing the N uptake capacity. Besides, the miR169-*NFYA* module shows a response to low PO_4_^3−^ availability in wheat. Overexpression of *TaNFYA*-*B1* significantly increases NO_3_^−^ and PO_4_^3−^ uptake at different nutrient supply levels by up-regulating the expression of NO_3_^−^ and PO_4_^3−^ transporters (Qu et al. 2015). The miR444-*MADS* module also follows the same pattern (Yan et al. 2014). Overall, miRNAs fine-tune plant N and Pi signaling transduction by influencing the expression of N and Pi transporters. However, the precise mechanisms by which miRNAs participate in the sensing and acquisition of N and Pi and directly regulate N and Pi transporters remains to be further explored.

#### MiRNAs involved in N and Pi signaling transduction mediated by hormones

miRNAs can regulate N and Pi signaling transduction by altering the biosynthesis of plant hormones. In *M*. *truncatula*, the expression level of miR160 is dependent on the Early Nodulin (ENOD) factor, and miR160 targets the auxin response factor (ARF) gene, which inhibits the expression of the Gretchen Hagen3 (GH3) gene, thereby reducing auxin levels and increasing lateral root numbers (Nizampatnam et al. 2015). mtr-miR160c and mtr-miR160d act on the peripheral region and primordium of the nodule, respectively, which is a piece of evidence that the miRNA family members mediate different spatio-temporal regulations (Bustos-Sanmamed et al. 2013). Besides, after prolonged NO_3_^−^ treatment, N metabolites from NO_3_^−^ assimilation increase miR393 expression but decrease auxin signaling F-box 3 (AFB3) expression. The similar root structure in *afb3* mutants and transgenic plants overexpressing miR393 confirms that miR393/AFB3 inhibits the growth of the main root but stimulates lateral root branching through the convergence of positive signals from external NO_3_^−^ and negative signals from internal N metabolites (Vidal et al. 2010). These investigations suggest that miRNAs fine-tune the synthesis of phytohormone and the formation of root system architecture (RSA). Further studies are needed to elucidate how miRNAs fine-tune multiple phytohormone pathways and RSA to enable plants to dynamically adapt to variable environments.

#### MiRNAs involved in N and Pi signaling transduction through integrating plant–microbe symbiosis

Plant species recruit soil microorganisms by regulating the N and Pi signals to enhance the defense against nutrient deficiency and improve the utilization of nutrient. The roots of flowering plants have physical and informational communications with mycorrhizae, which improve nutrient uptake and facilitate host adaptation to sophisticated and changeable external environments. For example, intrastromal mycelia extending from ectomycorrhizal (EM) root tips generally have a greater capacity to absorb inorganic N than non-mycorrhizal (NM) root tips under salt stress (Sa et al. 2019). Besides, a recent groundbreaking study reveals the central role of PHR1(-like) TFs in establishing plant-mycorrhizal symbiosis (Pazhamala and Giri 2023). The widespread recruitment of plant signals that are involved in different aspects of mycorrhizal symbiosis under PHR1/PHL controlling may underscore the importance of plant-mycorrhizae interaction in maintaining Pi homeostasis.

Intriguingly, miR171 family members (miR171a ~ h) are associated with the colonization of arbuscular mycorrhizal (AM) fungi. In *M*. *truncatula*, miR171h is induced to express in roots that are colonized by AM fungi and roots under high N and Pi, where it targets NSP2, an adjuster derived from SL which negatively regulates mycorrhizal symbiosis (Lauressergues et al. 2012; Liu et al. 2011). Other miR171 family members (miR171a, c-g) silence *MtLOM1*, a GRAS protein family ramification, almost in root cells (Couzigou et al. 2017), whereas miR171b targets *MtLOM1* in arbuscular mycorrhizal colonized root cells, thereby facilitating fungal colonization in the root cortex. This discrepancy is interrelated to the mismatch of the miR171b cleavage site that protects the *MtLOM1* transcript from degradation in arbuscular-containing cells (Couzigou et al. 2017). Thus, these studies demonstrate that miR171 family members contribute to the plant-mycorrhiza symbiosis. In addition, the expression of miR399 family members is negatively correlated with AM colonization in plants (Pandey et al. 2018; Xu et al. 2018), however, its concrete regulatory mechanism remains unclear so far. Particularly, miR393 is negatively regulated in arbuscular mycorrhiza, thereby releasing the inhibitory effect on the auxin signaling pathway and promoting the growth of arbuscular mycorrhiza (Etemadi et al. 2014). However, their roles in regulating AM colonization in roots and further integrating Pi signals to affect Pi uptake remain unknown.

Specifically, legumes establish symbiotic interactions with rhizobia through N-fixing nodules, where miRNA-mediated regulations rely on symbiosis-related genes and provide an additional layer of control in fine-tuning reprogramming events (Song et al. 2019). The dynamics of miRNAs-mediated regulation of legume-rhizobia symbiosis are summarized in a recent review in detail (Tiwari et al. 2021). Continued emerging information suggests the indispensable role of miRNAs in the post-transcriptional gene regulation (PTGS) of symbiotic N fixation is being investigated increasingly. Consequently, a plethora of information about miRNAs-mediated regulation in the N and Pi signaling transduction in plant–microbe symbiotic processes has been carved, however there are still large gaps that need to be filled.

#### MiRNAs involved in plant N and Pi signaling transduction co-evolution

Interestingly, a few miRNAs affect both N and Pi uptake. For example, the miR166-RICE DOF DAILY FLUCTUATIONS 1 (*RDD1*)-mediated regulation results in a diurnally oscillating expression pattern of *RDD1*, and overexpression of *RDD1* significantly increases both NH_4_^+^ and PO_4_^3−^ uptake in rice (Iwamoto and Tagiri 2016). This suggests that miRNAs are involved in the co-evolution of N and Pi signaling transduction. In Arabidopsis and rice, the *PHRs*-miR399-*PHO2*-*PHTs* module acts as a positive module of Pi uptake and transportation under Pi deficiency, and miR827, a pivotal component to low N, has parallel properties (Lin et al. 2013). Under low Pi, *PHR2* as a MYB-CC family member positively regulates the expression of miR827 in rice (Zhou et al. 2008). NLA contains an SPX domain that can coordinate with the E2 ligase PHO2 to regulate PHT1 degradation, which controls Pi metabolism. Increased abundance of miR827 under N starvation inhibits the expression of *NLA* genes and triggers a ubiquitination regulatory pathway that negatively regulates Pi transporters in rice (Lin et al. 2013; Park et al. 2014; Yue et al. 2017). A relevant report states the co-evolution of N and Pi signals is regulated by the NO_3_^−^-dependent Pi homeostasis regulatory module. Pi content increases by over five times in shoots of Arabidopsis *nla* mutant under low N and high Pi, which is interrelated to the aforesaid model (Kant et al. 2011). Combinedly, a growing body of evidence supports that miRNAs fine-tune the N and Pi signaling transduction co-evolution, which transforms an initially single nutrient-centric view into a more comprehensive view of nutritional homeostasis.

Additionally, although the sequences of miRNAs are conserved, their abundance, targets, and regulatory patterns change to varying degrees in the defense processes among plant species against N and Pi stresses. miRNAs have evolved diverse members with different roles and responses among different plant species. Thus, we summarized and compared the expression patterns of miRNAs in plant tissues in response to N and Pi stresses (Supplementary Table 1, and 2). More miRNAs that regulate N and Pi signaling transduction need to be further explored.

### N and Pi-related miRNAs as pleiotropic signals involved in diverse processes

The N and Pi signaling transduction affects not only the uptake and re-mobilization of N and Pi, but the production of metabolites and biosynthesis of oxidative radical scavengers. Apart from N and Pi stresses, other stress factors can also possibly activate the expression of N and Pi-related miRNAs. For example, under salt and drought stress, the MYB2 transcription factor binds to MBS elements in the miR399f promoter and enhances the miR399f promoter activity (Baek et al. 2013). Thus, as pleiotropic signals, miRNAs regulating the N and Pi signaling transduction are involved in diverse biological processes.

For instance, miR444a targets ANR1, a MADS-box transcription factor, which is a major component of the NO_3_^−^ signaling transduction. Under high NO_3_^−^ concentration conditions, overexpression of miR444a increased NO_3_^−^ transporter gene expression, thereby affecting plant adaptation to N-limiting conditions (Yan et al. 2014). And miR444 positively regulates brassinosteroids (BRs) biosynthesis by suppressing MADS-box, which directly represses the transcription of BR-deficient dwarf 1 (*OsBRD1*), a key BR biosynthetic gene. Recently, the NH_4_^+^ and BRs signaling crosstalk is required for NH_4_^+^-dependent inhibition of root elongation, in which NH_4_^+^ activates the miR444-*OsBRD1* module (Jiao et al. 2020). Moreover, the molecular cascade of miR444 and its targets MADS box (OsMADS23, OsMADS27A, and OsMADS57) directly control the transcription of RNA-dependent RNA polymerase 1 (*RDR1*), which is a key component of the antiviral RNA silencing pathway and is responsible for the production of virally activated endogenous siRNAs that stimulate broad-spectrum antiviral activity by silencing host genes (Wang et al. 2016). In the absence of viral infection, the expression of *OsRDR1* is inhibited by the MADS. Following viral infection, miR444 is induced to reduce the expression of MADS. The *OsRDR1*-dependent RNA silencing pathway is then activated to defend against rice virus infection by silencing viral RNA and host genes (Wang et al. 2016). Thus, the miR444-MADS module provides a switch for maintaining N homeostasis and activating immune processes. Besides, IAA-Ala Resistant3 (IAR3), as a target of miR167a, is required for the tolerance of plants to drought (Kinoshita et al. 2012). These investigations demonstrate that miRNAs regulating N and Pi signals exhibit multiple regulatory activities in diverse biological processes of plants.

### Characteristics of miRNAs as mobile signals in N and Pi signaling transduction

The systematic regulation of N and Pi acquisition and fixation in roots depends on the root signals, which are regulated by the soil conditions. It is worth noting that the expression of miR169, miR827, and miR2111 strongly depends on P or N status in the phloem of rapeseed (Pant et al. 2009), indicating they possibly act as mobile signals to regulate N and Pi signaling transduction. Besides, the effects of artificial miRNAs are evident outside the domains of their expression, suggesting that miRNAs can be transferred to adjacent cells, and it is first confirmed by the investigation of miR165/166 in Arabidopsis (Felippes et al. 2011). miR827 has also been verified to be transported in shoot-to-root under Pi starvation, whereas mi827* is non-mobile (Huen et al. 2017). miRNAs as signaling stimulators could move from one cell to another or over long distances to precisely regulate N and Pi signaling transduction, which is further highlighted and supported in the following cases.

#### MiR390 as a cell-to-cell signal in N signaling transduction

**Fig. 3:**
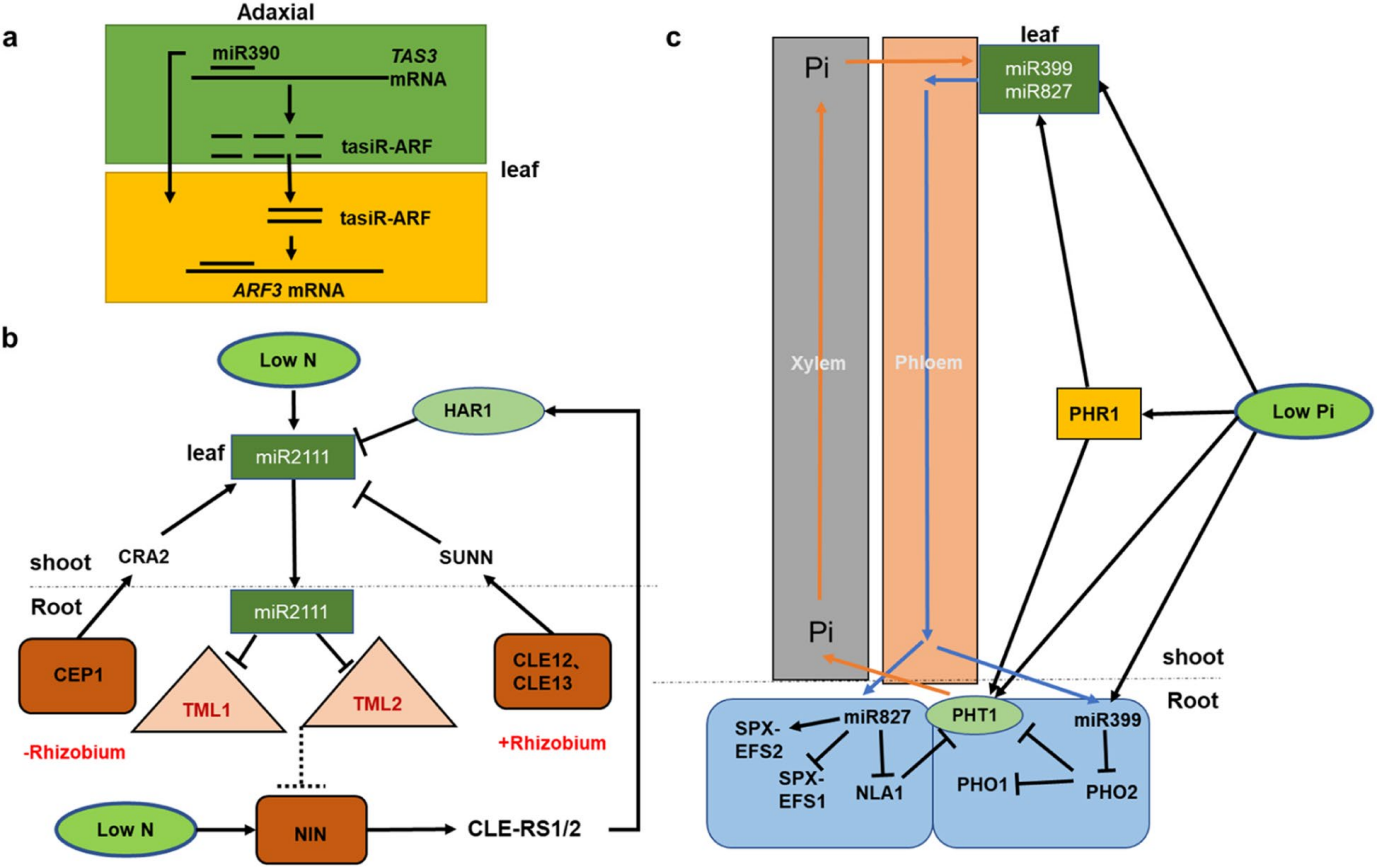
Mobile miRNAs that regulate N and Pi signaling transduction. **a** Mature miR390 and tasiR-ARFs act as mobile signals in Arabidopsis leaves and regulate N signaling transduction. **b** Low N induces miR2111 with mobility as a systemic effector involved in the regulation of the rhizobia symbiosis and N uptake. **c** Under low Pi conditions, the Pi signals are transported from the root to the stem through the phloem. Stem-derived miR399 and miR827 move to the roots to target PHO2, NLA, and E3 ligase, respectively, to increase Pi uptake. The black line with an arrow represents the positive regulation, the black line with a smooth top represents the negative regulation, and the dotted line needs further verification

In plants, miR390 directs *trans-acting siRNA3* (TAS3) transcripts through a dual targeting mode to produce tasiRNAs to regulate ARF genes (de Felippes et al. 2017; Marin et al. 2010), and silencing MtARF2/3/4a/4b changes the mRNA levels of nodule signaling pathway 2 (MtNSP2) (Kirolinko et al. 2021). These results suggest that miR390 regulates N signals. The miR390 precursors (*MIR390a* and *MIR390b*) are transcribed in the vasculature and pith beneath the shoot apical meristem of plants and are preferentially expressed on the anterior epidermis in developing leaves. Intriguingly, mature miR390 is present where its precursors accumulate and also in abaxial epidermis of leaves (Chitwood et al. 2009). Furthermore, there is a lack of concordance between the miR390 precursors and mature miR390 expression in maize shoot tips (Marin et al. 2010). Combinedly, these results strongly support that miR390 is a mobile molecule. Besides, AGO7 is specifically expressed on the abaxial epidermis of the Arabidopsis leaves and loaded with miR390 to target the TAS3 transcript. 21-nt tasiRNAs are then produced on the abaxial epidermis and move to the anterior epidermis of leaves and regionally inhibit the expression of the ARF3 transcripts (Fig. 3a). Although intercellular trafficking of tasiRNAs is more widely accepted than miRNAs, experiments support that the expression of miR390 and tasiR-ARFs is positively correlated in shoot apical meristems and leaf primordia. However, further explorations are required to demonstrate the mobility of miR390 and tasiR-ARF and the role of the movement in coordinating the N signaling transduction.

#### MiR2111 as a shoot–root-shoot signal in N signaling transduction

MiR2111, as a central long-distance effector in *M. truncatula* and *Lotus japonicus*, systematically regulates nodulation of the root system by targeting *TOO MUCH LOVE* (*TML*) and binding to inhomogeneous signal peptides and receptors (Gautrat et al. 2020; Okuma and Kawaguchi 2021; Sexauer et al. 2023). In the absence of rhizobia, the production of miR2111 induced by low N and C-terminally encoded peptide 1 (CEP1) signaling peptide is dependent on Compact Root Architecture 2 (CRA2) activity in the bud, thus favoring the nodulation ability of the root. The Super Numeric Nodule (SUNN) pathway negatively regulates miR2111 systemic effects when roots are nodulated (Fig. 3b) (Gautrat et al. 2020). Under N deficiency, CEP signal peptide in nitrogen-starved roots is transported to shoots via the CRA2 receptor, which positively regulates miR2111 and eventually facilitates plant nodulation (Mohd-Radzman et al. 2016). In the case of high N abundance, Clavata3/Embryo Surrounding Region 12 (CLE12) and Clavata3/Embryo Surrounding Region 13 (CLE13) signal peptides are produced in root nodules and can transfer to shoots through SUNN receptors and negatively regulate miR2111, thereby regulating nodule numbers (Mortier et al. 2010). Besides, recent studies have found that the MtNLP1-dependent N-induced MtCLE35 signaling peptide and SymCEP7 regulate nodulation through miR2111 (Ivanovici et al. 2023; Moreau et al. 2021). In summary, the regulatory modes are intricate shoot-to-root signaling transduction pathways.

#### MiR399 as a shoot-to-root and plant-to-plant signal in Pi signaling transduction

MiR399, a critical shoot-to-root signal in Arabidopsis, is demonstrated to maintain Pi homeostasis through phloem under low Pi (Rouached et al. 2010), and miR399* is also found to have the ability of shoot-to-root translocation (Huen et al. 2017). A similar circumstance has also been reported in common beans (Liu et al. 2010), which suggests the long-distance transportation of miR399 is conserved in plants (Nguyen et al. 2015) (Fig. 3c). Intriguingly, the transcript abundance of miR399 is increased in wild-type Arabidopsis plants adjacent to transgenic Arabidopsis overexpressing miR399 in the same hydroponic system (Betti et al. 2021), which suggests that miR399 is also transported between adjacent plants. Besides, the transportation of miR399 in phloem is presumably associated with RNA binding proteins due to the phloem SMALLRNA BINDING PROTEIN1 (*CmPSRP1*) regulating sRNA transportation in pumpkins (Yoo et al. 2004). Intriguingly, a recent study suggests that the movement of mature miR399f is independent of its biogenesis, sequence background, and length (Chiang et al. 2023). Thus, these above investigations reveal the molecular basis for the long-distance transport of miR399 in coordinating Pi signaling transduction. Together, miR399 acts as a shoot-to-root and plant-to-plant signal in Pi signaling transduction. However, identification of the nutrient-related mobile miRNAs, and validation of miRNA movement required in response to N and Pi stresses, and characterization of the mechanisms of translocation regulating miRNA movement under N and Pi stresses remains challenging.

### The RNA-RNA interactions affect miRNA-mediated N and Pi signaling transduction

Interestingly, the sophisticated RNA-RNA interactions, which are interdependent or antagonistic, can coordinate both NSR and PSR. Notably, ncRNAs often interact with miRNAs and co-regulate the expression of targets to modulate N and Pi signaling transduction (Fig. 1b). This adds to the regulatory complexity of N and Pi signaling transduction.

#### LncRNAs and miRNAs

Competing for endogenous RNAs hypothesis reveals a new RNA-RNA interaction mechanism, namely long noncoding RNAs (lncRNAs) affect miRNA-mediated gene silencing by competitively binding to miRNA (Salmena et al. 2011). Besides, lncRNAs have been found to serve as precursors of miRNAs to facilitate the cleavage of target genes (Song et al. 2021). Recently, genome-wide surveys of N-stress-responsive lncRNAs have been performed in poplar (Chen et al. 2016), maize (Lv et al. 2016), barley (Chen et al. 2020), and soybean (Golicz et al. 2018). A novel lncRNA T5120 modulated by NLP7 and NRT1.1, regulates N signals and improves the NUE in Arabidopsis (Liu et al. 2019), suggesting the important function of lncRNAs in the response of plants to N stress. Besides, the lncRNA-miRNA-mRNA networks involved in N metabolism are constructed in maize (Ma et al. 2021), rice (Shin et al. 2018), Moso Bamboo (Yuan et al. 2022), and poplar (Zhou et al. 2022). However, the role of the modules responding to low N stress has not been validated and further elucidated so far, and the gap needs to be addressed.

Moreover, *cis*-NATs, a class of lncRNAs, can respond to Pi stress in plants. In rice, *cis*-*NAT PHO1*;*2* promotes the translation of OsPHO1;2 and affects Pi homeostasis without altering the expression levels or nuclear export of *OsPHO1;2* mRNAs (Jabnoune et al. 2013). This result suggests that lncRNAs also have an important role in the response of plants to Pi stress. Different from the module of N, the interaction between lncRNAs and miRNA-mRNA module responding to low Pi has been demonstrated. The orthologs lncRNA At4 and INDUCED BY PI STARVATION 1 (IPS1) are Pi starvation-induced lncRNAs in Arabidopsis. The *At4*/*IPS1* family shares a 23-nt conserved motif that displays complementary sequences with miR399 and negatively regulates the transcript abundance of miR399 (Franco-Zorrilla et al. 2007). Under Pi starvation, the *At4* loss-of-function mutant accumulates excessive Pi in shoots, while plants overexpressing IPS1 have a lower content of Pi in shoots (Shin et al. 2006). The results suggest At4/IPS1 family-miR399-*PHO2* module plays a vital role in maintaining Pi homeostasis. Of course, mismatches in the targeted cleavage region of miR399 would hamper the complementarity to some extent. In maize, Pi deficiency-induced long noncoding RNA1 (*PILNCR1*) inhibits ZmmiR399-directed *ZmPHO2* cleavage. The *PILNCR1* abundance is significantly higher in the Pi-inefficient lines compared with the Pi-efficient lines, while the ZmmiR399 abundance is significantly higher in the Pi-efficient lines (Wang et al. 2023d). Besides, the lncRNA-miRNA-mRNA network is excavated using the Pi-sensitive genotype (Bogao) and the Pi-tolerant genotype (NN94156) (Lv et al. 2020). These findings propose the potential contributions of the network in regulating Pi signals of plants.

A positive correlation between the expression pattern of circRNAs and their host protein-coding genes is observed, suggesting that the circRNA-miRNA-mRNA network can modulate low N availability. Sequencing data also shows that miR399 is sponged by several novel circRNAs in soybean under Pi deficiency (Lv et al. 2020). However, so far, the expression profiles and predicted modules have only been constructed in wheat (Ren et al. 2018), soybean (Lv et al. 2020), moso bamboo (Zhu et al. 2023), and rape (Fu et al. 2023) under N and Pi deficiency. These results suggest the circRNAs-miRNAs-mRNAs network needs to be further explored to dissect the sophisticated regulatory mechanisms involved in N and Pi signaling transduction.

#### MiRNAs and siRNAs

MiRNAs can cleave not only protein-coding but noncoding transcripts, such as those from the PHAS loci and TAS loci, to induce phase secondary small interfering RNAs (phasiRNAs) and trans-acting secondary small interfering RNAs (tasiRNAs), respectively (Allen et al. 2005). The miR390-TAS3-ARF module is found in the nucleus part of the auxin-mediated network and assists lateral root (LR) growth (de Felippes et al. 2017; Fukuda et al. 2020; Marin et al. 2010). TAS3 is remarkably reduced under severe N deficiency (Fukuda et al. 2019), and the pathway is involved in promoting N-fixing capacity in *M. truncatula* (Kirolinko et al. 2021), suggesting that the pathway has a certain potential in response to N stress. Interestingly, bioinformatics analysis shows that NRT2.4, as a potential target of miR390-mediated TAS3-cleavage-derived siRNA, could promote N uptake in Arabidopsis (Fukuda et al. 2019). However, the module needs to be validated further. Besides, *ALTTAS3*, a short polyadenylated TAS3 subtype, can be regarded as a target mimicry to sequestrate miR390 due to the lack of a tasiRNA-producing region and retention of miR390 non-cleavable binding sites, and its function in N signaling transduction is unexplained (Traubenik et al. 2020). Overall, the role of miRNAs targeting multiple genes to trigger more siRNAs in N and Pi signaling transduction remains unclear. It is worth noting that uncommon sRNA-miRNA modules can also have unique effects on plants. A relevant report suggests that miR2118 regulates the reproductive capacity of a photosensitive male sterile mutant in rice by targeting *PMS1T*, a 21-nt phasiRNA precursor, under long daylight conditions (Fan et al. 2016). These investigations provide critical information for understanding the role of miRNAs and siRNAs in N and Pi signaling transduction.

## Application of the miRNA-mediated regulation: manipulation of miRNAs to maintain N and Pi homeostasis

Intensive research into the regulation of N and Pi signaling transduction has identified numerous N and Pi signals and allows the test of their potential to improve the NUE and PUE of plants and the tolerance of plants to N and Pi stresses. The manipulation of the expression of *MIR* genes and miRNAs opens the way to address the effects of fine-tuning N and Pi signals in maintaining N and Pi homeostasis of plants. Here, we provide a brief overview of some of the well-studied miRNAs and widely-used methods and techniques that might be effectively used for this strategy. These results inform the breeding targets, methods, and techniques of using more subtle strategies.

### Candidate miRNAs used to maintain N and Pi homeostasis

**Fig. 4:**
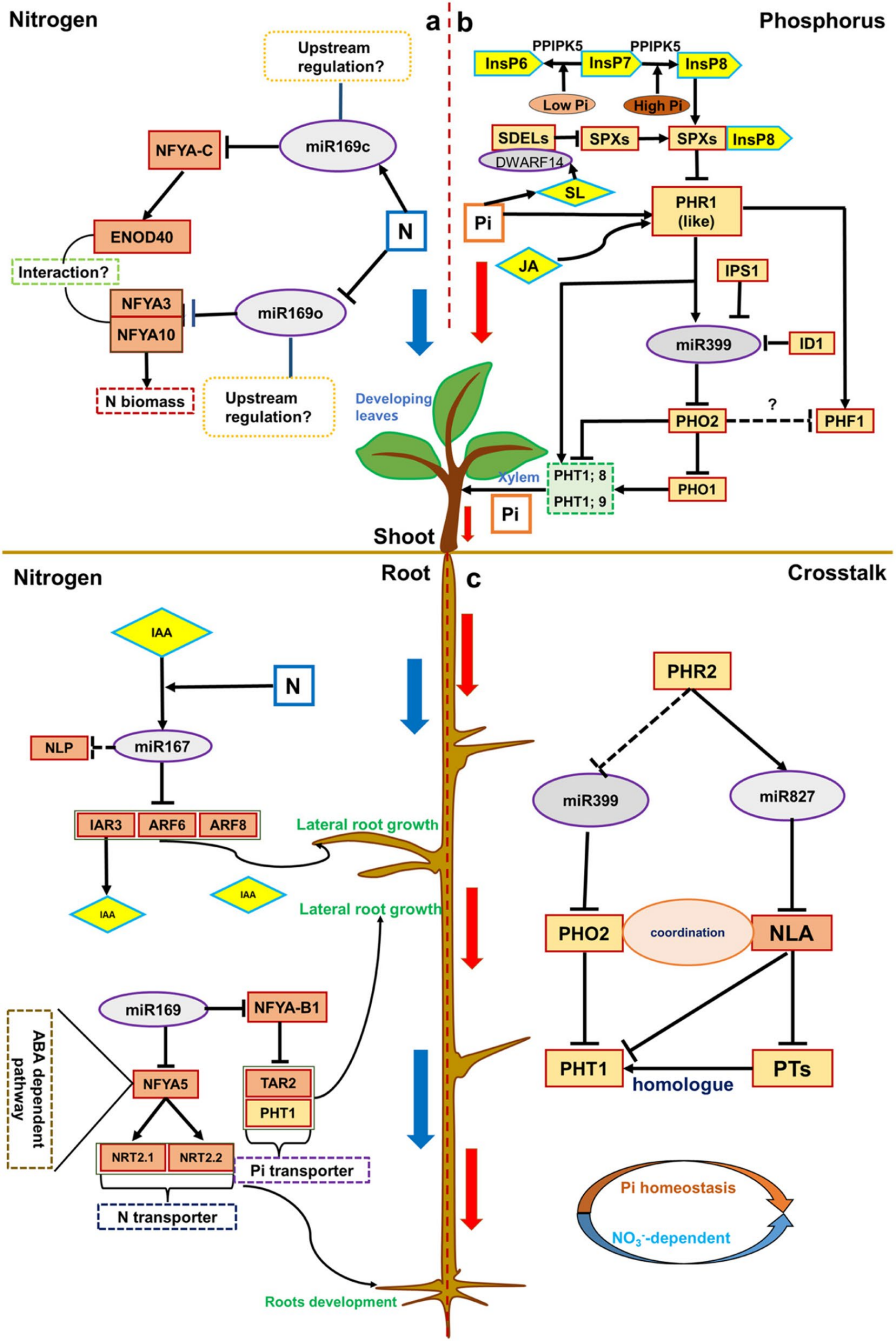
Well-studied miRNAs that maintain N and Pi homeostasis. **a** Regulations of miR167 and miR169 in maintaining N homeostasis. miR167 is a positive N signal. miR169a and miR169c are negative N signals, and miR169o is a positive N signal. **b** Regulation of miR399 in maintaining Pi homeostasis and the upstream regulation of miR399. **c** A co-evolution model of NO_3_^−^-dependent Pi homeostasis contributes to the maintenance of N and Pi homeostasis in plants. The black line with an arrow represents the positive regulation, and the black line with a smooth top represents the negative regulation

Several well-studied miRNAs maintaining N and Pi homeostasis are candidates that could be further utilized in the plant breeding. The molecular and physiological roles of the auxin-mediated miR167-ARF/IAR3 module, the miR169-NFYA-ENOD/NRT module, and the miR399-PHO2 module are compared and discussed below (Fig. 4).

#### MiR167: a positive N signal

MiR167 is first studied in Arabidopsis and targets *ARF6* and *ARF8*. The expression level of miR167 is decreased in roots of Arabidopsis under N stress, which triggers the accumulation of ARF8 in the pericytes and lateral root caps, and thereby stimulates the auxin signal and promotes lateral root formation and N absorption (Gifford et al. 2008). miR167a targets IAR3 that hydrolyzes an inactive form of auxin and releases bioactive auxin, suggesting miR167a-IAR3 module is involved in the N-signaling network by regulating the content of IAA in Arabidopsis (Kinoshita et al. 2012). In cotton, miR167a and miR167b are predicted to target *NLP* genes, and these genes are significantly upregulated, which can positively regulate the tolerance of plants to N deficiency (Ru et al. 2006; Wu et al. 2006). In soybean, the expression of miR167c is up-regulated in the vasculatures, pericytes, and cortex of soybean roots after inoculation with *Bradyrhizobium*. miR167c positively regulates the nodule numbers by repressing *GmARF8a* and *GmARF8b* (Wang et al. 2015b; Wu et al. 2006). Furthermore, auxin stimulates and suppresses the expression of miR167 and its targets, respectively. The regulation of miR167 on nodule initiation is dependent on the Nod factor receptor, which acts upstream of the nodulation-associated genes including nodule inception, nodulation signaling pathway1, early nodulin40-1, *NF-YA1* (previously known as HAEM activator), and *NF-YA2* (Wang et al. 2015b). Hence, the auxin-mediated miR167-ARF/IAR3 module positively regulates the N homeostasis.

#### MiR169: a complicated N signal

In Arabidopsis, the miR169 family contains 14 members, among which, miR169a is the main contributor to N uptake (Zhao et al. 2011). miR169a impairs the N uptake system as a negative regulator by inhibiting the expression of *NFYA5* in Arabidopsis. In addition, *NFYA5,* a positive regulator of AtNRT2.1 and AtNRT1.1, plays a central role in NO_3_^−^ transportation and is involved in the ABA-dependent pathway under N deficiency (Zhao et al. 2011). Furthermore, overexpression of *TaNFYA-B1*, a low-N inducible *NFYA* transcription factor that is negatively regulated by miR169, enhances the expression of auxin biosynthetic genes and nutrient transporters (NRT and PHT1), and significantly promotes lateral root growth and grain yield by increasing N uptake in wheat (Qu et al. 2015). In rice, *OsNF*-*YA5*, a target of osa-miR169a, directly enhances the expression of *OsNRT1.1A* and N uptake rate under N deficiency (Seo et al. 2023). These results suggest miR169a-*NFYA*-*B1* module may control a sophisticated N signaling transduction. Consistently, overexpression of miR169c displays a significant reduction in height, leaf size, root length, and nodule number of soybean. A regulatory pathway of miR169c-GmNFYA-C-GmENOD40 in response to N availability is discovered in soybean (Xu et al. 2021). Interestingly, transgenic rice overexpressing miR169o has more biomass accumulation with significantly increased NO_3_^−^ levels and total amino acid contents (Yu et al. 2018). Thus, miRNA family members may perform different functions. Furthermore, in maize, a prolonged NO_3_^−^ depletion may repress the miR169i/j/k transcription and induce the post-transcriptional expression of NFYA5, thereby altering the root development (Trevisan et al. 2012). Taken together, miR169 is one of the most core signals regulating N homeostasis.

#### MiR399: a positive Pi signal

In Arabidopsis and rice, roots perceive Pi-deficiency signals and induce the synthesis of miR399, which degrades the *PHO2* transcript by binding at five complementary sites located in the 200–400 bp upstream of 5' untranslated region (5' UTR). Transgenic Arabidopsis overexpressing miR399 promotes the Pi accumulation by contributing to the loss of *PHO2* encoding E2 ubiquitin binding enzyme. Additionally, the *IPS1* gene acts as a “miRNA target mimicry” by binding to the complementary region of miR399 to negatively regulate the miR399-*PHO2* pathway, which further increases the complexity of the Pi module in wheat (de Souza Campos et al. 2019). Besides, the expression of the *PHO2* gene is also directly modulated by PHR1 (Sega et al. 2020). Interestingly, PHR1 can directly up-regulate the miR399 transcript expression under PO_4_^3−^ starvation as a nuclear regulator of the miR399 gene promoter (Rouached et al. 2010). PHR1 can also be modulated by a small ubiquitin-like modifier (SUMO) E3 ligase (SIZ1), whose expression is up-regulated by Pi deficiency. In maize, INDETERMINATE1 (ID1), a regulator of floral transition, inhibits the expression of miR399 by directly binding to the promoter of the miR399 gene, which alleviates the inhibition of miR399 on the expression of the *PHO2* gene and ultimately contributes to maintaining Pi homeostasis. Interestingly, unlike the miR399 gene induced by PO_4_^3−^ deficiency, the expression of ID1 is not affected by the external inorganic orthophosphate state, indicating that ID1 is an autonomous regulator of Pi homeostasis (Wang et al. 2023c). Notably, the NO_3_^−^-dependent co-evolution model of Pi homeostasis cooperatively maintains N and Pi homeostasis of plants (Fig. 4c). Overall, miR399 is a core and positive Pi signal in diverse plants.

### Widely-used research methods/technologies of miRNAs for accelerating plant breeding

**Fig. 5:**
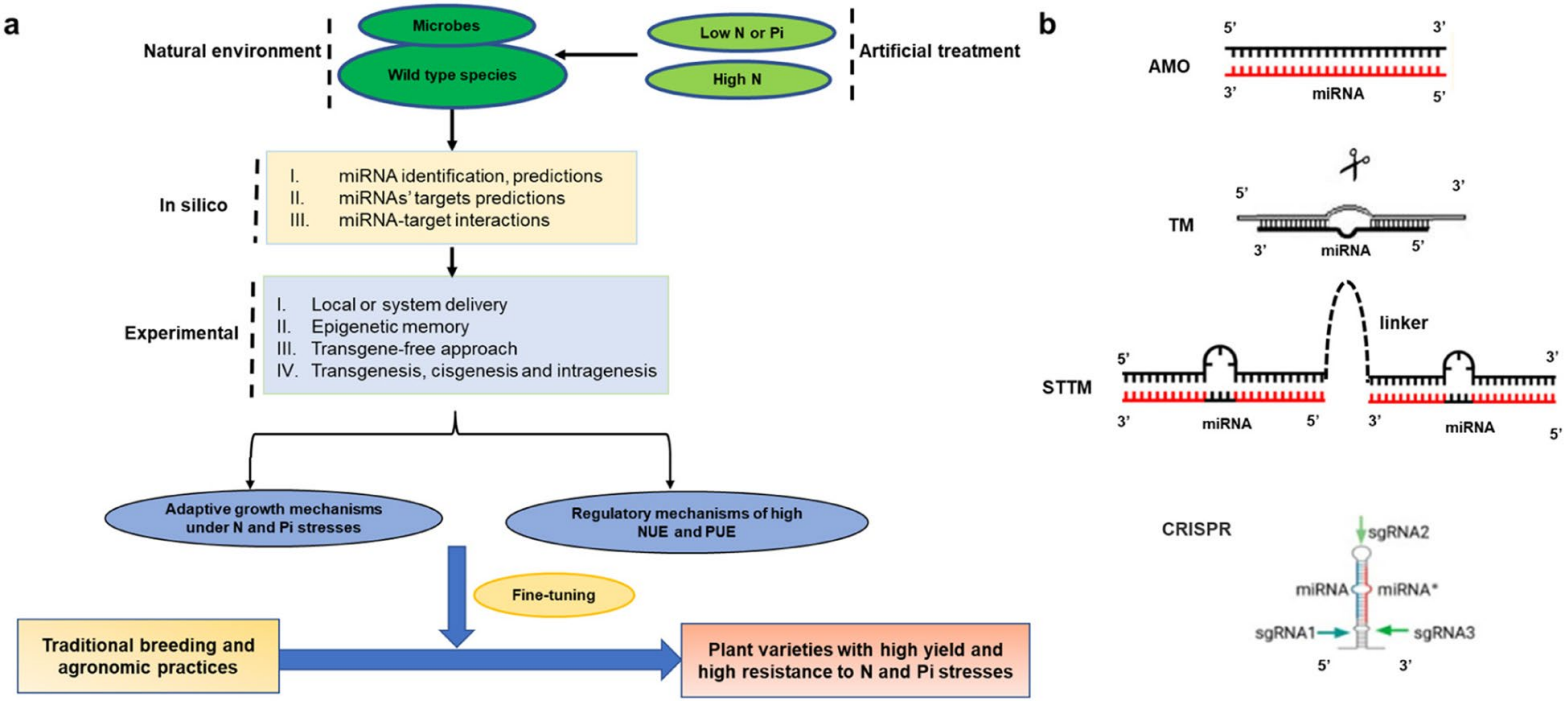
Widely-used methods and technologies for accelerating plant breeding via fine-tuning *MIR* genes and miRNAs. **a** The methods might be feasible to accelerate plant breeding via fine-tuning *MIR* genes and miRNAs. The miRNAs-target modules that maintain N and Pi homeostasis are identified through the methods described in this review. **b** The technologies of regulating miRNA expression in transgenic research. Exogenous modeling of anti-microRNA oligonucleotides (AMO) is used. Target mimic (TM), short tandem target mimic (STTM), and CRISPR/Cas system are widely-used in fine-tuning *MIR* genes and miRNAs

The function of miRNAs in maintaining N and Pi homeostasis can be explored by fine-tuning the *MIR* genes or miRNAs using transgenic, cisgenic, and intrageneric methods, which was summarized in Fig. 5a. Plant species that naturally exist in the surrounding rhizosphere microbes could be used to explore the genetic differences of plants. Plant and microbial genome data could be considered parallelly. Additionally, the plant species subjected to artificial nutrient stress, and multi-omics analysis of plants and microbes could be conducted. Bioinformatics analysis websites and tools are listed in Supplementary Table 3, which support more miRNA-target modules to be scooped out. Totally, these methods provide comprehensive insights into the influence of miRNAs in regulating the NUE and PUE of plants and the tolerance of plants to N and Pi stresses.

Currently, the physiological and molecular verification and application of transgenic plants represents the most critical step for the implementation of basic research and the advancement of sustainable agriculture. Thus, we focus on discussing the technologies for fine-tuning miRNAs in transgenic research in the following (Fig. 5b).

#### Increase and decrease of miRNA expression

Straightforward technologies of manipulating miRNA expression are the overexpression of *MIR* genes, target mimic (TM), and short tandem target mimic (STTM), all driven by artificially selected promoters (Basso et al. 2019). Overexpression of *MIR* genes increases the expression of miRNAs and enhances their functions. Besides, exogenous modeling of anti-microRNA oligonucleotides (AMO) produces complementary sequences of miRNAs and bind to the miRNAs, which is used in the TM and STTM technologies. Overexpression of TM and STTM reduces the expression of miRNAs and attenuates their functions by competitively binding to them. Notably, the effects of these techniques on miRNA expression are limited. Besides, an inappropriate promoter might lead to undesirable pleiotropic effects. Researchers should choose suitable promoters according to the requirements, including constitutive promoters, such as the 35S promoters, and inducible promoters activated only under certain conditions, such as the root-forming or nodule-forming promoters and the N-stress-responsive or Pi-stress-responsive promoters.

#### Elimination of miRNA expression

Meanwhile, the clustered regularly interspaced short palindromic repeats CRISPR and CRISPR/Cas system modifying DNA sequences have revolutionized biotechnology and provided genetic tools for genetically modified plants (Manghwar et al. 2020). The CRISPR/Cas system is increasingly becoming one of the most popular tools for biotechnology applications due to its accuracy, efficiency, and cost-effectiveness (Pan et al. 2023), and is widely used in the genome editing of model plants and crops, such as Arabidopsis, rice (*Oryza sativa*), wheat (*Triticum aestivum*), tomato (*Lycopersicon esculentum*) (Bi et al. 2020; Lv et al. 2024; Miao et al. 2019; Zhang et al. 2021). A successful example of CRISPR/Cas9 editing applied to improve the PUE is recently reported (Miller et al. 2022). The newly created CRISPR/Cas9 reagent is used to target mutagenesis of *PHO2*, the target of miR399, which generated transgenic alfalfa (*Medicago sativa* L*.*) plants with super accumulated Pi levels 3- to sixfold higher than that of wild-type.

So far, there are limitations and opportunities in the application of CRISPR/Cas system in miRNA editing breeding. The knockout is mainly based on the canonical or discontinued SpCas9 that can induce *cis*-acting element mutations in the promoter region of nutrient-sensitive miRNAs. For instance, knockout of miR396 elevates GROWTH REGULATING FACTOR transcripts (*OsGRF4* and *OsGRF8*) in the corresponding rice variants (Lin et al. 2021). And not all *MIR* genes can be modified via the CRISPR system with SpCas9, which requires the 5'-NGG PAM sites for the further cleavage. Moreover, not all plants are transformed efficiently for evolutionary research and the transgenic plants are usually limited to particular genotypes (Li et al. 2019; Pan et al. 2023; Wang et al. 2019). Encouragingly, various plasmid delivery systems have been established to overcome the limitation imposed by genotypes, such as nanoparticles (NPs), PEG-mediated protoplast transformation, and haploid inducer-mediated gene editing systems (Kelliher et al. 2019), which bring opportunities to promote the miRNA editors breeding. Overall, CRISPR/Cas-mediated miRNA editing breeding is a promising and feasible genetic engineering technology to maintain N and Pi homeostasis.

Efficient *MIR* gene editing is achieved by miRNA-target complexes induced by small and medium indels, substitutions, and base editing mismatches (Sretenovic et al. 2021). Emerging gene editing tools, such as the AFID system that can generate predictable polynucleotide deletions (Wang et al. 2020b), may be alternative tools for plant miRNA editing. CRISPR activation (CRISPRa) and CRISPR interference (CRISPRi) systems are also emerging techniques for modulating miRNA expression in plants (Manghwar et al. 2019; Russell et al. 2024). In conclusion, fine-tuning the *MIR* genes or miRNAs may be a promising area of future research to maintain N and Pi homeostasis.

## Conclusions and future perspective

Plants have evolved miRNA-target modules to regulate the tolerance to nutrient stress, some of which are evolutionarily related to environmental adaptation. A single miRNA may target more than one transcript, and vice versa, to fine-tune the expression of genes, which converges into a sophisticated and extremely fault-tolerant crosstalk. Accumulating findings highlight that miRNAs furnish a bridge for the transportation and stockpile of N and Pi nutrition in the plants, plant-environment interactions, and plant-plant communications through the modulation of N and Pi signaling transduction.

The main themes that emerged from previous studies are the role of miRNAs in enhancing NUE and PUE of plants, as well as the adaptive responses of plants to N and Pi stresses. Destructive effects of nutrient stress on plants are largely dependent on the inheritance and variation of plants and the influence of the environment due to the immobility of plants and the complexity of the environment. One area of future research that needs to be addressed in more detail concerns the function of miRNAs in the communications between plants and the environment. For example, the role of plant symbionts in exporting sRNA to induce transboundary gene silencing to improve N and Pi homeostasis of plants has not been well reported and requires further investigation. In addition, future studies on miRNA*s as underappreciated regulatory components would also expand our understanding of *MIR* genes in regulating plant N and Pi homeostasis.

The research directions mainly focus on the upstream regulatory mechanisms and the downstream regulatory roles of miRNAs responding to nutrient stress. In particular, the upstream regulatory mechanisms of miRNAs are still in their infancy, such as the mechanisms by which miRNAs are loaded for precise regulation. For instance, in Arabidopsis, Pi starvation significantly increases the expression of miR778, miR827, and miR2111, and inhibits the expression of miR168, miR395, and miR398. Interestingly, the expression of miR778 and miR2111 quickly decrease by approximately two folds within three hours of the restored Pi supply (Paul et al. 2015). This phenomenon is related to the higher number of Pi-responsive motifs in the *cis*-acting region of the *MIR* gene promoter compared with non-responsive *MIR* genes (Zeng et al. 2010). Plant epigenetic modification regulators, including DNA methylation, may also regulate miRNA expression and thereby maintain cell survival under nutrient stress. Therefore, the network of miRNAs, targets, DNA methylation, and metabolic factors is also an important direction for future research.

Fine-tuning *MIR* genes or miRNAs is a powerful biotechnology strategy to acquire high-yield crop varieties with sustainable economic and ecological benefits. Well-studied miRNAs and widely-used methods and techniques provide the basis for the strategy. Particularly, recent developments in genome editing are gradually being used to engineer miRNAs and the targets. CRISPR/Cas9 can be used for the negative regulation of miRNA expression to breed plants with high NUE and PUE and strong tolerance to N and Pi stresses. Alternatively, mutations in the *cis*-regulatory regions of *MIR* genes can be induced to regulate their expression. Notably, the accumulation or reduction of miRNAs regulating the N and Pi signaling transduction may produce unexpected phenotypes in transgenic plants due to the pleiotropy. Researchers should have a comprehensive knowledge framework to critically utilize the research achievements. Undoubtedly, we believe that future research surrounding miRNAs would provide additional insights into solving high-profile problems, such as excessive application of inorganic fertilizers, unbalanced absorption of plant nutrients, and the decline of biodiversity.

## Supplementary Information

Supplementary Material 1.

## Data Availability

All the data supporting the claims contained in this manuscript are provided in the submission and can be shared publicly after acceptance of the manuscript for publication by Stress Biology.

## Abbreviations

N: Nitrogen
Pi: Phosphorus
MiRNA: MicroRNA
NcRNA: Non-coding RNA
TF: Transcription factor
NUE: N use efficiency
PUE: Pi use efficiency
PNR: Primary nitrate response
NSR: N starvation response
PS: Pi starvation
PSR: Pi starvation rescue
LncRNA: Long non-coding RNA
SiRNA: Small interfering RNA
CircRNA: Circular RNA
NO_3_^−^: Nitrate
NH_4_^+^: Ammonium
NRT1/PTR: Nitrate transporter 1/peptide transporter family
NRT2: Nitrate transporter 2 family
CLC: Chloride channel family
NLP: NIN-LIKE PROTEIN
ROS: Reactive oxygen species
Ca^2+^: Calcium
PHT: High-affinity transporter
PHR: Phosphorus starvation response
PHO: Phosphate
PHF: Phosphate transporter facilitator
InsP: Inositol polyphosphate
ENOD: Early Nodulin
ARF: Auxin response factor
BR: Brassinosteroid
IAA: Indoleacetic acid
IAR3: IAA-Ala Resistant3
NFYA: Nuclear Factor-Y Subunit A
IPS: Induced by Pi Starvation
PhasiRNA: Phased small interfering RNA
TasiRNA: Trans-acting small interfering RNA
TM: Target mimic
STTM: Short tandem target mimic
CRISPR: Clustered regularly interspaced short palindromic repeat
