# Supplementary material for: MicroRNAs as potent regulators in nitrogen and phosphorus signaling transduction and their applications
Source: Stress Biol. 2024 Sep 12;4(1):38. doi: 10.1007/s44154-024-00181-x (PMC11393275; doi:10.1007/s44154-024-00181-x)
Supplement: Supplementary file 1 — Supplementary Material 1. [file 44154_2024_181_MOESM1_ESM.doc]

# Supplemental information

# MicroRNAs as Potent Regulators in Nitrogen and Phosphorus Signaling Transduction and Their Applications

**Yuzhang Yang, Yanting Liang, Chun Wang and Yanwei Wang***

## Supplemental tables

Supplemental Table 1 Different miRNAs under N limitation with their target genes, and tissue specific responses in different plants

| MiRNA family | Target genes | Tissue expression | Species | Reference |
| --- | --- | --- | --- | --- |
| miR156 | *SPL* | R (Up) | Maize | (Zhao et al. 2012) |
|  |  | R (Up) | *Arabidopsis* | (Liang et al. 2012) |
|  |  | R (Up) | Wheat | (Zhao et al. 2015) |
| miR159 | *MYB*,  *TCP* | R (Up) | Maize | (Zhao et al. 2012) |
|  |  | PS (Dw) | *Brassica napus* | (Pant et al. 2009) |
|  |  | WP (Dw) | *Populus tomentosa* | (Chen et al. 2015) |
| miR160 | *ARF* | R (Up) | Maize | (Xu et al. 2011) |
|  |  | R (Up) | *Arabidopsis* | (Liang et al. 2012) |
|  |  | R (Dw) | Wheat | (Sinha et al. 2015) |
| miR162 | *DCL* | R (Up) S (Up) | Maize | (Zhao et al. 2012) |
| miR164 | *NAC*,  *CUC* | L (Up) R (Dw) S (Dw) | Maize | (Xu et al. 2011)  (Zhao et al. 2012) |
|  |  | R (Dw) | Rice | (Nischal et al. 2012) |
|  |  | R (Dw) | Wheat | (Sinha et al. 2015; Zuluaga et al. 2018) |
|  |  | R (Up) | Wheat | (Zuluaga et al. 2018) |
| miR166 | *HD-ZIP* | R(Dw) | Maize | (Trevisan et al. 2012) |
|  |  | R(Dw) | Wheat | (Zuluaga et al. 2018) |
| miR167 | *ARF* | R (Dw) | Maize | (Xu et al. 2011) |
|  |  | R (Up) S (Up) | Maize | (Zhao et al. 2012) |
|  |  | R (Dw) | *Arabidopsis* | (Liang et al. 2012) |
|  |  | R (Dw) L(Dw) | *Phaseolus vulgaris* | (Valdes-Lopez et al. 2010) |
|  |  | R (Dw) | Wheat | (Sinha et al. 2015) |
| miR168 | *AGO1* | R (Dw) | Maize | (Xu et al. 2011) |
| miR169 | *HAP2*,  *C/bf*,  *NFYA* | R (Dw) S (Dw) SD (Dw) | *Arabidopsis* | (Liang et al. 2012;  Pant et al. 2009) |
|  |  | R (Dw) S (Dw) L (Dw) | Maize | (Zhao et al. 2012;  Xu et al. 2011) |
|  |  | R (Dw) S (Dw) | Soybean | (Wang et al. 2013) |
|  |  | PS (Dw) | *Brassica napus* | (Pant et al. 2009) |
|  |  | R (Dw) L (Dw) | *Phaseolus vulgaris* | (Valdes-Lopez et al. 2010) |
|  |  | R (Dw) S (Dw) L (Dw) | Wheat | (Hou et al. 2020;  Zuluaga et al. 2017) |
| miR171 | *SCL* | R (Up) S (Dw) | *Arabidopsis* | (Liang et al. 2012) |
|  |  | R (Up) S (Up) | Maize | (Zhao et al. 2012) |
|  |  | R (Dw) S (Dw) | Soybean | (Wang et al. 2013) |
| miR172 | *AP2-like* | L (Up) S (Up) | Maize | (Zhao et al., 2012;  Xu et al. 2011) |
|  |  | R (Dw) | *Arabidopsis* | (Liang et al. 2012) |
| miR319 | *TCP* | L (Up) | Wheat | (Zuluaga et al. 2017) |
|  |  | R (Dw) | Maize | (Xu et al. 2011) |
|  |  | R (Dw) S (Dw) | Soybean | (Wang et al. 2013) |
|  |  | R (Dw) L (Dw) | *Phaseolus vulgaris* | (Valdes-Lopez et al. 2010) |
| miR393 | Auxin receptors | R (Up) | Maize | (Zhao et al. 2012) |
|  |  | WP (Up) | *Populus tomentosa* | (Chen et al. 2015) |
|  |  | R (Dw) L (Dw) | Wheat | (Zuluaga et al. 2017) |
| miR394 | *F box* | S (Up) | Maize | (Zhao et al. 2012) |
|  |  | R (Dw) S (Dw) | Soybean | (Wang et al. 2013) |
| miR395 | ATP Sulfurylase,  Sulfate transporters | R (Dw) | *Arabidopsis* | (Liang et al. 2012) |
|  |  | R (Dw) | Maize | (Zhao et al. 2012) |
| miR396 | *GRF* | R (Up/Dw) S (Up/Dw) | Soybean | (Wang et al. 2013) |
|  |  | R (Dw) | Maize | (Zhao et al. 2012) |
|  |  | L (Up) | *Phaseolus vulgaris* | (Valdes-Lopez et al. 2010) |
| miR397 | *LAC* | L (Dw) S (Dw) R (Dw) | Maize | (Zhao et al. 2012;  Xu et al. 2011) |
|  |  | R (Dw) | *Arabidopsis* | (Liang et al. 2012) |
|  |  | R (Dw) S (Dw) | Soybean | (Wang et al. 2013) |
| miR398 | *COX5b,*  *CSD* | R (Dw) SD (Dw) | *Arabidopsis* | (Liang et al. 2012;  Pant et al. 2009) |
|  |  | R (Dw) L (Dw) S (Dw) | Maize | (Zhao et al. 2012;  Xu et al. 2011) |
|  |  | R (Dw) S (Dw) | Soybean | (Wang et al. 2013) |
| miR399 | *E2/UBC24* | PS (Up) | *Brassica napus* | (Pant et al. 2009) |
|  |  | L (Dw) R (Dw) | Maize | (Xu et al. 2011;  Zhao et al. 2012) |
|  |  | L (Dw) R (Dw) | Wheat | (Zhao et al. 2015) |
|  |  | R (Dw) | *Arabidopsis* | (Liang et al. 2012) |
|  |  | R (Dw) L (Dw) | *Phaseolus vulgaris* | (Valdes-Lopez et al. 2010) |
| miR408 | *PCY*,  *LAC* | L (Dw) R (Dw) S (Dw) | Maize | (Trevisan et al. 2012;  Xu et al. 2011;  Zhao et al. 2012) |
|  |  | R (Dw) | *Arabidopsis* | (Liang et al. 2012) |
|  |  | R (Dw) S (Dw) | Soybean | (Wang et al. 2013) |
|  |  | R (Dw) L (Dw) | *Phaseolus vulgaris* | (Valdes-Lopez et al. 2010) |
| miR444 | *MADS box* | R (Up) | Rice | (Yan et al. 2014) |
|  |  | R (Up) L (Up) | Wheat | (Zhao et al. 2015) |
| miR528 | *POD, SOD* | L (Dw) R (Dw) S (Dw) | Maize | (Trevisan et al. 2012;  Xu et al. 2011;  Zhao et al. 2012) |
|  |  | R (Dw) | Rice | (Nischal et al. 2012) |
| miR780 | Na+/H+ antiporter | R (Up) | *Arabidopsis* | (Liang et al. 2012) |
| miR826 | *AOP2* | R (Up) | *Arabidopsis* | (Liang et al. 2012) |
| miR827 | *NLA* | R (Up) L (Up） | Wheat | (Zuluaga et al. 2018) |
|  |  | R (Dw) | *Arabidopsis* | (Liang et al. 2012) |
|  |  | L (Dw) R (Dw) S (Dw) | Maize | (Xu et al. 2011;  Zhao et al. 2012) |
|  |  | L (Dw) | Wheat | (Zuluaga et al. 2018) |
| miR857 | *LAC* | R (Dw) | *Arabidopsis* | (Liang et al. 2012) |
| miR1133 | *CML,*  *SET domain,*  *ENODLs* | R (Dw) | Wheat | (Zhao et al. 2015) |

Abbreviations: *SPL*, SQUAMOSA PROMOTER BINDING PROTEIN-LIKE; *MYB*, MYELOBLASTOSIS; *TCP*, TEOSINTE BRANCHED1/CYCLOIDEA/PROLIFERATING CELL FACTORS; *ARF*, AUXIN RESPONSE FACTORS; *DCL*, Dicer like proteins; *NAC*, NO APICAL MERISTEM/ARABIDOPSIS TRANSCRIPTION ACTIVATION FACTOR/CUP-SHAPED COTYLE; *CUC*, CUP-SHAPED COTYLEDON; *HD-ZIP*, HOMEODOMAIN-LEUCINE ZIPPER; *AGO1*, ARGONAUTE1; *HAP2*, Hook-Associated Protein 2; *C/bf*, CAAT BINDING FACTOR; *NFYA*, NUCLEAR FACTOR Y SUBUNIT A; *SCL*, SCARECROW-LIKE TRANSCRIPTION FACTORS; AP2-like, AP2-LIKE TRANSCRIPTION FACTORS; *F box*, F-box protein; *GRF*, GROWTH REGULATING FACTOR; *LAC*, LACCASE; *COX5b*, CYTOCHROME C OXIDASE SUBUNIT 5b; *CSD*, COMPLIMENTARY SEX DETERMINER; *E2/UBC24*, UBIQUITIN-CONJUGATING (E2) ENZYME 24; *PCY*, PLANTACYANIN; *MADS box*, MADS-box TRANSCRIPTION FACTORS; *POD*, PEROXIDASE; *SOD*, SUPER OXIDE DIMUTESE; *AOP2*, ALKEBYL HYDROXALKYL PRODUCING 2; *NLA*, nitrogen limitation adaptation; *CML*, Calmodulin-like protein; *SET* *domain*, (Su(var)3-9, Enhancer-of-zeste, Trithorax) domain; *ENODLs*, Early nodulin-like proteins. Plant tissue: R, root; L, leaf; S, shoot; SD, seedling; PS, Phloem sap; WP, Whole plantlets; ‘Up’ represents that the expression of miRNAs is up-regulated under N deficiency; ‘Dw’ represents that the expression of miRNAs is down-regulated under N deficiency.

Supplemental Table 2 Different miRNAs under Pi limitation with their target genes and tissue specific responses in different plants

| MiRNA family | Target genes | Tissue expression | Species | Reference |
| --- | --- | --- | --- | --- |
| miR156 | *SPL* | R (Up) | *Arabidopsis* | (Hsieh et al. 2009) |
|  |  | R (Up) L (Dw) | White lupin | (Zhu et al. 2010) |
| miR157 | *SPL* | N (Up) | Common bean | (Valdes-Lopez et al. 2010) |
| miR159 | *MYB*, *TCP* | R (Up) SM (Dw) L (Dw) | White lupin | (Zhu et al. 2010) |
|  |  | R (Up) | Soybean | (Zeng et al. 2010) |
| miR160 | *ARF* | R (Up) L (Dw) | White lupin | (Zhu et al. 2010) |
|  |  |  |  |  |
| miR164 | *NAC* | R (Up) SM (Dw) L (Dw) | White lupin | (Zhu et al. 2010) |
| miR166 | *HD-ZIP* | R (Up) SM (Dw) L (Dw) | White lupin | (Zhu et al. 2010) |
| miR167 | *ARF* | R (Up) L (Dw) | White lupin | (Zhu et al. 2010) |
| miR168 | *AGO1* | R (Up) L (Up) | White lupin | (Zhu et al. 2010) |
| miR169 | *HAP2*, *NFYA* | SD (Dw) R (Dw) S (Dw) | *Arabidopsis* | (Hou et al. 2020; Hsieh et al. 2009; Pant et al. 2009) |
| miR171 | *SCL* | SM (Up) L (Up) | White lupin | (Zhu et al. 2010) |
| miR172 | *AP2-like* | L (Dw) | Tomato | (Gu et al. 2010) |
| miR319 | *TCP* | R (Up) SM (Dw) | White lupin | (Zhu et al. 2010) |
|  |  | R (Up) L (Dw) | Tomato | (Gu et al. 2010) |
|  |  | R (Dw) | Soybean | (Zeng et al. 2010) |
| miR390 | *ARF* | R (Dw) | White lupin | (Zhu et al. 2010) |
| miR394 | *F-box* | R (Up) | Tomato | (Gu et al. 2010) |
| miR395 | ATP sulfurylase, sulfate  transporters | R (Dw) SM (Up) L (Up) | White lupin | (Zhu et al. 2010) |
|  |  | R (Dw) S (Dw) | *Arabidopsis* | (Hsieh et al. 2009) |
| miR396 | *GFR* | R (Up) L (Dw) | White lupin | (Zhu et al. 2010) |
| miR397 | *LAC* | L (Dw) | White lupin | (Zhu et al. 2010) |
|  |  | L (Dw) | common bean | (Valdes-Lopez et al. 2010) |
| miR398 | COX5b-1, CCS1-COX | L (Up) | Tomato | (Gu et al. 2010) |
|  |  | SD (Dw) S (Dw) R (Dw) | *Arabidopsis* | (Hsieh et al. 2009) |
|  |  | R (Dw) | Soybean | (Zeng et al. 2010) |
|  |  | L (Dw) | Common bean | (Valdes-Lopez et al. 2010) |
| miR399 | *UBC24* | SD (Up) R (Up) S (Up) | *Arabidopsis* | (Chiou et al. 2006; Hsieh et al. 2009; Lundmark et al. 2010) |
|  |  | R (Up) L (Up) | *Medicago* *truncatula* | (Branscheid et al. 2010) |
|  |  | R (Up) L (Up) | Common bean | (Liu et al. 2010) |
|  |  | L (Up) | White lupin | (Zhu et al. 2010) |
|  |  | R (Up) L (Up) | Tomato | (Gu et al. 2010) |
|  |  | R (Up) S (Up) | Rice | (Zhou et al. 2008) |
|  |  | S (Up) | Barley | (Hackenberg et al. 2013) |
| miR444 | *MADS-box* | R (Up) | Rice | (Yan et al. 2014) |
| miR778 | *SET domain* | SD (Up) R (Up) S (Up) | *Arabidopsis* | (Hou et al. 2020; Hsieh et al. 2009; Pant et al. 2009) |
| miR827 | *NLA* | SD (Up) R (Up) S (Up) | *Arabidopsis* | (Hou et al. 2020; Hsieh et al. 2009; Lundmark et al. 2010; Pant et al. 2009) |
|  |  | R (Up), S (Up) | Rice | (Lin et al. 2010) |
|  |  | S (Up) | Barley | (Hackenberg et al. 2013) |
| miR828 | *TAS4* | S (Up) | *Arabidopsis* | (Hsieh et al. 2009) |
| miR2111 | *TML* | SD (Up) R (Up) S (Up) | *Arabidopsis* | (Hou et al. 2020; Hsieh et al. 2009; Pant et al. 2009) |

Abbreviations: *SPL*, SQUAMOSA promoter binding protein-like; *MYB*, v-myb avian myeloblastosis viral oncogene homolog; *TCP*, Teosinte branched1/Cincinnata/proliferating; *ARF*, auxin response factor; *NAC*, NAM, ATAF1/2, and CUC2; *HD-ZIP*, HOMEODOMAIN-LEUCINE ZIPPER; *AGO1*, ARGONAUTE1; *HAP2*, Hook-Associated Protein 2; *NFYA*, NUCLEAR FACTOR Y SUBUNIT A; *SCL*, SCARECROW-LIKE TRANSCRIPTION FACTORS; AP2-like, AP2-LIKE TRANSCRIPTION FACTORS; *F box*, F-box protein; *F box*, F-box protein; *GFR*, growth Regulating Factor; *LAC*, LACCASE; *COX5b*, CYTOCHROME C OXIDASE SUBUNIT 5b; *E2/UBC24*, UBIQUITIN-CONJUGATING (E2) ENZYME 24; *MADS box*, MADS-box TRANSCRIPTION FACTORS; *SET domain*, (Su(var)3-9, Enhancer-of-zeste, Trithorax) domain; *NLA*, nitrogen limitation adaptation; *TAS*, *trans-*acting siRNA; *TML*, *Too Much Love*. Plant tissue: R, root; L, leaf; S, shoot; SD, seedling; PS, Phloem sap; WP, Whole plantlets; ‘Up’ represents that the expression of miRNAs is up-regulated under N deficiency; ‘Dw’ represents that the expression of miRNAs is down-regulated under N deficiency.

**Supplemental Table 3 Comparison of plant miRNAs and their targets identification related da**tabase

| Database type | Name | Brief function description | Web link | Reference |
| --- | --- | --- | --- | --- |
| Comprehensive database | PMRD | Plant miRNA Database. | <http://bioinformatics.cau.edu.cn/PMRD/> | (Zhang et al. 2010) |
| miRBase | The most influential miRNA database. | [http://www.mirbase.org](http://www.mirbase.org/) | (Kozomara et al. 2019) |
| PmiREN | A comprehensive functional plant miRNA database. | <https://pmiren.com/database> | (Guo et al. 2020) |
| sRNAanno | MiRNAs, phased small interfering RNAs (phased siRNAs or phasiRNAs), and heterochromatic siRNAs (hc-siRNAs); 138 plant species with sequenced genomes and sRNA-seq datasets. | <http://plantsrnas.org/> | (Chen et al. 2021) |
| Rfam | Rfam contains information about all types of noncoding RNA (ncRNA) families. Over 3000 miRNA families in different organisms. | <https://rfam.xfam.org/> | (Kalvari et al. 2018) |
| MiRNA-target interaction database | MTide | The identification of miR-target interaction in plants. | <http://bis.zju.edu.cn/MTide/> | (Zhang et al. 2015) |
| miRTarBase | The experimentally validated miR-target interactions database. | <http://mirtarbase.mbc.nctu.edu.tw/index.php> | (Li et al. 2013) |
| PlantMirnaT | A miRNA-mRNA integrated analysis system. | <https://sites.google.com/site/biohealthinformaticslab/resources> | (Rhee et al. 2015) |
| BioVLAB-MMIA-NGS | MiRNA and mRNA integrated analysis using high-throughput sequencing data. | <http://epigenomics.snu.ac.kr/biovlab_mmia_ngs/> | (Chae et al. 2015) |
| TAPIR | Target prediction for Plant miRs. | <http://bioinformatics.psb.ugent.be/webtools/tapir/> | (Rost et al. 2015) |
| PMTED | Expression profiles of miRNA targets in the plethora of existing microarray data; Variable information of targets. | <https://ngdc.cncb.ac.cn/databasecommons/database/id/4632> | (Sun et al. 2013) |
| TarBase | The indexing of experimentally supported miRNA targets. Over 1 million entries corresponding to around 670 000 unique miRNA-target pair interactions, which are supported by more than 33 experimental methodologies. | [https://bio.tools/tarbase#!](https://bio.tools/tarbase#!) | (Huang et al. 2019) |
| C-mii | A tool for plant miR and target identification. | <http://www.biotec.or.th/isl/c-mii> | (Numnark et al. 2012) |
| Semirna | Searching for plant miRNAs using target sequences. | <http://www.bioinfocabd.upo.es/semirna/> | (Muñoz-Mérida et al. 2012) |
| MiRNA annotations in specific species | miSolRNA | MiRNAs and their predicted targets mainly in fruit development model plant, tomato. | <http://www.misolrna.org/> | (Bazzini et al. 2010) |
| mirtronDB | All type of information on mirtrons such as sequences, structure, publications, etc., is included. specially describes mirtrons in chordates, vertebrates and plants. | <http://mirtrondb.cp.utfpr.edu.br/> | (Yu et al. 2019) |
| MepmiRDB | A specific database for miRNAs in medicinal plants. | <http://mepmirdb.cn/mepmirdb/index.html> | (Da Fonseca et al. 2019) |
| PmiRExAt | Plant miRNA expression in multiple tissues and developmental stages, includes wheat, rice, maize and *Arabidopsis*. | <http://www.pmirexat.nabi.res.in/> | (Gurjar et al. 2016) |
| ASRP | A collection of different type small RNAs, NGS datasets of sRNAs, PARE and mutants of key genes in small RNA biogenesis pathway in model plant. | <https://ngdc.cncb.ac.cn/databasecommons/database/id/1255> | (Gustafson, 2004) |
| PmiRKB | The miRNAs of two model plants, *Arabidopsis* and rice. Four major functional modules such as SNPs, Pri-miR, MiR–Tar, Self-reg. | <https://ngdc.cncb.ac.cn/databasecommons/database/id/603> | (Meng et al. 2010) |
| Specific related miRNA annotations | PlanTE-MIR | Database for transposable element-related plant microRNAs. | <http://bioinfo-tool.cp.utfpr.edu.br/plantemirdb/> | (R. Lorenzetti et al. 2016) |
| PASmiR | A solid platform for collection, standardization, and searching of these miRNA-stress regulation data in plants. | <https://ngdc.cncb.ac.cn/databasecommons/database/id/4426> | (Zhang et al. 2013) |
| Some tools | miTRATA | A tool for miRNA truncation and tailing analysis. | <https://wasabi.dbi.udel.edu/~apps/ta/> | (Patel et al. 2016) |
| miRanalyzer | MiRNA detection and analysis tool for next-generation sequencing experiments. | <http://bioinfo5.ugr.es/miRanalyzer/miRanalyzer.php> | (Hackenberg et al. 2009) |
| miRPlant | An integrated tool for identification of plant miRNA from RNA sequencing Data. | <http://www.australianprostatecentre.org/research/software/mirplant> | (An et al. 2014) |
| P-SAMS | A web tool for artificial miRNAs and synthetic trans-acting small interfering RNAs. | [http://p-sams.carringtonlab.org](http://p-sams.carringtonlab.org/) | (Fahlgren et al. 2016) |

## Supplemental References

An J, Lai J, Sajjanhar A, Lehman ML, and Nelson CC (2014) MiRPlant: an integrated tool for identification of plant miRNA from RNA sequencing data. BMC Bioinformatics 15:275. https://doi.org/10.1186/1471-2105-15-275

Bazzini AA, Asis R, Gonzalez V, Bassi S, Conte M, Soria M, Fernie AR, Asurmendi, S, and Carrari, F (2010) MiSolRNA: a tomato microRNA relational database. BMC Plant Biol 10:240. https://doi.org/10.1186/1471-2229-10-240

Branscheid A, Sieh D, Pant BD, May P, Devers EA, Elkrog A, Schauser L, Scheible WR, and Krajinski F (2010) Expression pattern suggests a role of MiR399 in the regulation of the cellular response to local Pi increase during arbuscular mycorrhizal symbiosis. Mol Plant Microbe Interact 23:915-926. https://doi.org/10.1094/MPMI-23-7-0915

Chae H, Rhee S, Nephew KP, and Kim S (2015) BioVLAB-MMIA-NGS: MicroRNA-mRNA integrated analysis using high-throughput sequencing data. Bioinformatics 31:265-267. https://doi.org/10.1093/bioinformatics/btu614

Chen C, Li J, Feng J, Liu B, Feng L, Yu X, Li G, Zhai J, Meyers BC, and Xia R (2021) SRNAanno-a database repository of uniformly annotated small RNAs in plants. Hortic Res 8. https://doi.org/10.1038/s41438-021-00480-8

Chen M, Bao H, Wu Q, and Wang Y (2015) Transcriptome-wide identification of miRNA targets under nitrogen deficiency in Populus tomentosa using degradome sequencing. Int J Mol Sci 16:13937-13958. https://doi.org/10.3390/ijms160613937

Chiou T, Aung K, Lin S, Wu C, Chiang S, and Su C (2006) Regulation of phosphate homeostasis by microRNA in Arabidopsis. Plant Cell 18:412-421. <https://doi.org/10.1105/tpc.105.038943>

Da Fonseca BHR, Domingues DS, and Paschoal AR (2019) MirtronDB: a mirtron knowledge base. Bioinformatics 35:3873-3874. https://doi.org/10.1093/bioinformatics/btz153

Fahlgren N, Hill ST, Carrington JC, and Carbonell A (2016) P-SAMS: a web site for plant artificial microRNA and synthetic trans-acting small interfering RNA design. Bioinformatics 32:157-158. https://doi.org/10.1093/bioinformatics/btv534

Gu M, Xu K, Chen A, Zhu Y, Tang G, and Xu G (2010) Expression analysis suggests potential roles of microRNAs for phosphate and arbuscular mycorrhizal signaling in Solanum lycopersicum. Physiol Plant 138:226-237. <https://doi.org/10.1111/j.1399-3054.2009.01320.x>

Guo Z, Kuang Z, Wang Y, Zhao Y, Tao Y, Cheng C, Yang J, Lu X, Hao C, and Wang T, et al (2020) PmiREN: a comprehensive encyclopedia of plant miRNAs. Nucleic Acids Res 48:D1114-D1121. https://doi.org/10.1093/nar/gkz894

Gurjar AKS, Panwar AS, Gupta R, and Mantri SS (2016) PmiRExAt: Plant miRNA expression atlas database and web applications. Database 2016:w60. https://doi.org/10.1093/database/baw060

Gustafson AM (2004) ASRP: the Arabidopsis small RNA project database. Nucleic Acids Res 33:D637-D640. https://doi.org/10.1093/nar/gki127

Hackenberg M, Shi BJ, Gustafson P, and Langridge P (2013) Characterization of phosphorus-regulated miR399 and miR827 and their isomirs in barley under phosphorus-sufficient and phosphorus-deficient conditions. BMC Plant Biol 13:214. https://doi.org/10.1186/1471-2229-13-214

Hackenberg M, Sturm M, Langenberger D, Falcón-Pérez JM, and Aransay AM (2009) MiRanalyzer: a microRNA detection and analysis tool for next-generation sequencing experiments. Nucleic Acids Res. 37:W68-76. https://doi.org/10.1093/nar/gkp347

Hou G, Du C, Gao H, Liu S, Sun W, Lu H, Kang J, Xie Y, Ma D, and Wang C (2020) Identification of microRNAs in developing wheat grain that are potentially involved in regulating grain characteristics and the response to nitrogen levels. BMC Plant Biol 20. https://doi.org/10.1186/s12870-020-2296-7

Hsieh L, Lin S, Shih AC, Chen J, Lin W, Tseng C, Li W, and Chiou T (2009) Uncovering small RNA-Mediated responses to phosphate deficiency in Arabidopsis by deep sequencing. Plant Physiol 151:2120-2132. https://doi.org/10.1104/pp.109.147280

Huang H, Lin Y, Li J, Huang K, Shrestha S, Hong H, Tang Y, Chen Y, Jin C, Yu Y, et al (2019) MiRTarBase 2020: Updates to the experimentally validated microRNA-target interaction database. Nucleic Acids Res 48:D148-D154. https://doi.org/10.1093/nar/gkz896

Kalvari I, Nawrocki EP, Argasinska J, Quinones-Olvera N, Finn RD, Bateman A, and Petrov AI (2018) Non-Coding RNA analysis using the rfam database. Curr Protoc Bioinformatics 62:e51. https://doi.org/10.1002/cpbi.51

Kozomara A, Birgaoanu M, and Griffiths-Jones S (2019) MiRBase: From microRNA sequences to function. Nucleic Acids Res 47:D155-D162. https://doi.org/10.1002/cpbi.51

Li J, Liu S, Zhou H, Qu L, and Yang J (2013) StarBase v2.0: decoding miRNA-ceRNA, miRNA-ncRNA and protein-RNA interaction networks from large-scale CLIP-Seq data. Nucleic Acids Res 42:D92-D97. https://doi.org/10.1093/nar/gkt1248

Liang G, He H, and Yu D (2012) Identification of nitrogen starvation-responsive microRNAs in Arabidopsis thaliana. PLoS One 7:e48951. https://doi.org/10.1371/journal.pone.0048951

Lin SI, Santi C, Jobet E, Lacut EEl Kholti N, Karlowski WM, Verdeil JL, Breitler JC, Perin C, Ko SS, et al (2010) Complex regulation of two target genes encoding SPX-MFS proteins by rice miR827 in response to phosphate starvation. Plant Cell Physiol 51:2119-2131. https://doi.org/10.1093/pcp/pcq170

Liu J, Allan DL, and Vance CP (2010) Systemic signaling and local sensing of phosphate in common bean: cross-talk between photosynthate and microRNA399. Mol Plant 3:428-437. https://doi.org/10.1093/mp/ssq008

Lundmark M, Korner CJ, and Nielsen TH (2010) Global analysis of microRNA in Arabidopsis in response to phosphate starvation as studied by locked nucleic acid-based microarrays. Physiol Plant 140:57-68. https://doi.org/10.1111/j.1399-3054.2010.01384.x

Meng Y, Gou L, Chen D, Mao C, Jin Y, Wu P, and Chen M (2010) PmiRKB: a plant microRNA knowledge base. Nucleic Acids Res 39:D181-D187. https://doi.org/10.1093/nar/gkq721

Muñoz-Mérida A, Perkins JR, Viguera E, Thode G, Bejarano ER, and Pérez-Pulido AJ (2012) Semirna: searching for plant miRNAs using target sequences. OMICS 16:168-177. https://doi.org/10.1089/omi.2011.0115

Numnark S, Mhuantong W, Ingsriswang S, and Wichadakul D (2012) C-mii: a tool for plant miRNA and target identification. BMC Genomics 13 Suppl 7:S16. https://doi.org/10.1186/1471-2164-13-S7-S16

Nischal L, Mohsin M, Khan I, Kardam H, Wadhwa A, Abrol YP, Iqbal M, and Ahmad A (2012) Identification and comparative analysis of microRNAs associated with low-N tolerance in rice genotypes. PLoS One 7:e50261. https://doi.org/10.1371/journal.pone.0050261

Pant BD, Musialak-Lange M, Nuc P, May P, Buhtz A, Kehr J, Walther D, and Scheible W (2009) Identification of nutrient-responsive Arabidopsis and rapeseed microRNAs by comprehensive real-time polymerase chain reaction profiling and small RNA sequencing. Plant Physiol 150:1541-1555. <https://doi.org/10.1104/pp.109.139139>

Patel P, Ramachandruni SD, Kakrana A, Nakano M, and Meyers BC (2016) MiTRATA: a web-based tool for microRNA truncation and tailing analysis. Bioinformatics 32:450-452. https://doi.org/10.1093/bioinformatics/btv583

R Lorenzetti AP, A De Antonio GY, Paschoal AR, and Domingues DS (2016) PlanTE-MIR DB: a database for transposable element-related microRNAs in plant genomes. Funct Integr Genomic 16:235-242. https://doi.org/10.1007/s10142-016-0480-5

Rhee S, Chae H, and Kim S (2015) PlantMirnaT: miRNA and mRNA integrated analysis fully utilizing characteristics of plant sequencing data. Methods 83:80-87. https://doi.org/10.1016/j.ymeth.2015.04.003

Rost HL, Rosenberger G, Aebersold R, and Malmstrom L (2015) Efficient visualization of high-throughput targeted proteomics experiments: TAPIR. Bioinformatics 31:2415-2417. https://doi.org/10.1093/bioinformatics/btv152

Sinha SK, Rani M, Bansal N, Gayatri Venkatesh K, and Mandal PK (2015) Nitrate starvation induced changes in root system architecture, carbon:nitrogen metabolism, and miRNA expression in Nitrogen-Responsive wheat genotypes. Appl Biochem Biotechnol 177:1299-1312. https://doi.org/10.1007/s12010-015-1815-8

Sun X, Dong B, Yin L, Zhang R, Du W, Liu D, Shi N, Li A, Liang Y, and Mao L (2013) PMTED: a plant microRNA target expression database. BMC Bioinformatics 14:174. https://doi.org/10.1186/1471-2105-14-174

Trevisan S, Nonis A, Begheldo M, Manoli A, Palme K, Caporale G, Ruperti B, and Quaggiotti S (2012) Expression and tissue-specific localization of nitrate-responsive miRNAs in roots of maize seedlings. Plant Cell Environ 35:1137-1155. https://doi.org/10.1111/j.1365-3040.2011.02478.x

Valdes-Lopez O, Yang SS, Aparicio-Fabre R, Graham PH, Reyes JL, Vance CP, and Hernandez G (2010) MicroRNA expression profile in common bean (Phaseolus vulgaris) under nutrient deficiency stresses and manganese toxicity. New Phytol 187:805-818. https://doi.org/10.1111/j.1469-8137.2010.03320.x

Wang Y, Zhang C, Hao Q, Sha A, Zhou R, Zhou X, and Yuan L (2013) Elucidation of miRNAs-mediated responses to low nitrogen stress by deep sequencing of two soybean genotypes. PLoS One 8:e67423. https://doi.org/10.1371/journal.pone.0067423

Xu Z, Zhong S, Li X, Li W, Rothstein SJ, Zhang S, Bi Y, and Xie C (2011) Genome-wide identification of microRNAs in response to low nitrate availability in maize leaves and roots. PLoS One 6:e28009. https://doi.org/10.1371/journal.pone.0028009

Yan Y, Wang H, Hamera S, Chen X, and Fang R (2014) MiR444a has multiple functions in the rice nitrate-signaling pathway. Plant J 78:44-55. https://doi.org/10.1111/tpj.12446

Yu, D, Lu, J, Shao, W, Ma, X, Xie, T, Ito, H, Wang, T, Xu, M, Wang, H, and Meng, Y (2019) MepmiRDB: a medicinal plant microRNA database. Database (Oxford) 2019. https://doi.org/10.1093/database/baz070

Zeng HQ, Zhu YY, Huang SQ, and Yang ZM (2010) Analysis of phosphorus-deficient responsive miRNAs and cis-elements from soybean (Glycine max L.). J Plant Physiol 167:1289-1297. https://doi.org/10.1016/j.jplph.2010.04.017

Zhang S, Yue Y, Sheng L, Wu Y, Fan G, Li A, Hu X, Shangguan M, and Wei C (2013) PASmiR: a literature-curated database for miRNA molecular regulation in plant response to abiotic stress. BMC Plant Biol 13:33. https://doi.org/10.1186/1471-2229-13-33

Zhang Z, Jiang L, Wang J, Gu P, and Chen M (2015) MTide: an integrated tool for the identification of miRNA-target interaction in plants. Bioinformatics 31:290-291. https://doi.org/10.1093/bioinformatics/btu633

Zhang Z, Yu J, Li D, Zhang Z, Liu F, Zhou X, Wang T, Ling Y, and Su Z (2010) PMRD: plant microRNA database. Nucleic Acids Res 38: D806-D813. https://doi.org/10.1093/nar/gkp818

Zhao M, Tai H, Sun S, Zhang F, Xu Y, and Li WX (2012) Cloning and characterization of maize miRNAs involved in responses to nitrogen deficiency. PLoS One 7:e29669. https://doi.org/10.1371/journal.pone.0029669

Zhao Y, Guo L, Lu W, Li X, Chen H, Guo C, and Xiao K (2015) Expression pattern analysis of microRNAs in root tissue of wheat (Triticum aestivum L.) under normal nitrogen and low nitrogen conditions. J Plant Biochem Biotechnol 24:143-153

Zhou J, Jiao F, Wu Z, Li Y, Wang X, He X, Zhong W, and Wu P (2008) OsPHR2 is involved in Phosphate-Starvation signaling and excessive phosphate accumulation in shoots of plants Plant Physiol 146:1673-1686. https://doi.org/10.1104/pp.107.111443

Zhu YY, Zeng HQ, Dong CX, Yin XM, Shen QR, and Yang ZM (2010) MicroRNA expression profiles associated with phosphorus deficiency in white lupin (Lupinus albus L). Plant Sci 178:23-29. https://doi.org/10.1016/j.plantsci.2009.09.011

Zuluaga DL, De Paola D, Janni M, Curci PL, and Sonnante G (2017) Durum wheat miRNAs in response to nitrogen starvation at the grain filling stage. PLoS One 12:e183253. https://doi.org/10.1371/journal.pone.0183253

Zuluaga DL, Liuzzi V, Curci PL, and Sonnante G (2018) MicroRNAs in durum wheat seedlings under chronic and short-term nitrogen stress. Funct Integr Genomics 18:645-657. https://doi.org/10.1007/s10142-018-0619-7
